# Angiogenesis regulators S100A4, SPARC and SPP1 correlate with macrophage infiltration and are prognostic biomarkers in colon and rectal cancers

**DOI:** 10.3389/fonc.2023.1058337

**Published:** 2023-02-21

**Authors:** Elena Kazakova, Militsa Rakina, Tatiana Sudarskikh, Pavel Iamshchikov, Anna Tarasova, Liubov Tashireva, Sergei Afanasiev, Alexei Dobrodeev, Lilia Zhuikova, Nadezhda Cherdyntseva, Julia Kzhyshkowska, Irina Larionova

**Affiliations:** ^1^ Laboratory of Translational Cellular and Molecular Biomedicine, Tomsk State University, Tomsk, Russia; ^2^ Cancer Research Institute, Tomsk National Research Medical Center, Russian Academy of Sciences, Tomsk, Russia; ^3^ Laboratory of Genetic Technologies, Siberian State Medical University, Tomsk, Russia; ^4^ Institute of Transfusion Medicine and Immunology, Institute for Innate Immunoscience (MI3), Medical Faculty Mannheim, University of Heidelberg, Mannheim, Germany; ^5^ German Red Cross Blood Service Baden-Württemberg – Hessen, Mannheim, Germany

**Keywords:** colorectal cancer, tumor-associated macrophage, SPP1, S100A4, SPARC, angiogenesis, chemotherapy, prognosis

## Abstract

**Introduction:**

Increasing evidence suggests that it is necessary to find effective and robust clinically validated prognostic biomarkers that can identify “high-risk” colorectal cancer (CRC) patients. Currently, available prognostic factors largely include clinical-pathological parameters and focus on the cancer stage at the time of diagnosis. Among cells of tumor microenvironment (TME) only Immunoscore classifier based on T lymphocytes showed high predictive value.

**Methods:**

In the present study, we performed the complex analysis of mRNA and protein expression of crucial regulators of tumor angiogenesis and tumor progression, expressed by tumor-associated macrophages (TAMs): S100A4, SPP1 and SPARC. Colon and rectal cancer patients were investigated independently and in a combined cohort (CRC). For mRNA expression, we analyzed RNA sequencing data obtained from TCGA (N=417) and GEO (N=92) cohorts of colorectal cancer patients. For protein expression, we performed IHC digital quantification of tumor tissues obtained from 197 patients with CRC treated in the Department of abdominal oncology in Clinics of Tomsk NRMC.

**Results:**

High S100A4 mRNA expression accurately predicted poor survival for patients with CRC independently of cancer type. SPARC mRNA level was independent prognostic factors for survival in colon but not in rectal cancer. SPP1 mRNA level had significant predictive value for survival in both rectal and colon cancers. Analysis of human CRC tissues revealed that S100A4, SPP1 and SPARC are expressed by stromal compartments, in particular by TAMs, and have a strong correlation with macrophage infiltration. Finally, our results indicate that chemotherapy-based treatment can change the predictive direction of S100A4 for rectal cancer patients. We found that S100A4 stromal levels were higher in patients with better response to neoadjuvant chemotherapy/chemoradiotherapy, and S100A4 mRNA levels predicted better DFS among non-responders.

**Discussion:**

These findings can help improve the prognosis of patients with CRC based on S100A4, SPP1 and SPARC expression levels.

## 1 Introduction

Colorectal cancer (CRC) is the third most common malignancy and the second-leading cause of cancer death in the world due to the unmet screening programs, therapeutic strategies, and increasing incidence rates (1). Colorectal cancer is characterized by high inter- and intra-tumoral heterogeneity (2, 3). Although colorectal cancer is a more general and widely used term, there is still a differentiation into two distinct localizations: colon cancer (CC) and rectal cancer (RC). There are evidences accumulated towards considering CC and RC as self-standing tumor entities due to their topography, surgical challenge, therapy, complications, and relapse patterns (4, 5). CRC heterogeneity determines difficulties in choosing anticancer treatment, and also poses an obstacle in reaching therapeutic complete response (6). Although the response rate to systemic chemotherapies goes up to 50%, nearly all patients with CRC develop drug resistance, which limits the therapeutic efficacy of anticancer drugs and ultimately leads to chemotherapy failure (7, 8). These difficulties pose a demand for biomarker discovery that will help in improving treatment efficiency, early detection, and could be of value as diagnostic or prognostic markers.

Tumor microenvironment (TME), which consists of stromal and immune cells, has an essential role in tumor development (9). The key component of innate immunity in the TME is tumor-associated macrophages (TAMs) (10). TAMs regulate tumor growth by supporting cancer cell survival and proliferation, angiogenesis, and metastasis, as well as the response of cancer cells to therapeutic intervention (11). Angiogenesis is a basic process that provides the tumor with crucial nutrients and oxygen (12). The main pro-angiogenic regulator in tumors is VEGF (12). Despite the growing list of FDA-approved anti-VEGF drugs, the success of anti-angiogenic therapy is limited. Failure in VEGF-targeted therapy can be explained by the switching on the alternative pro-angiogenic activators (13). Recently collected data demonstrated that TAMs can be essential sources for plenty of novel angiogenesis-related proteins that belong to S100A class, SEMA family, chitinase-like proteins, growth factors, and proteins regulating cell-matrix interactions, etc (13). Among them, pro-angiogenic factors S100A4 and osteopontin (OPN, or SPP1) as well as anti-angiogenic factor SPARC, which have drawn our attention, since we have recently identified the deregulation of their expression in TAMs under chemotherapy exposure *in vitro* (unpublished data).

In the present study, for the first time we performed the complex analysis of mRNA and protein expression of S100A4, SPP1 and SPARC. We establish their prognostic significance in terms of survival rates and clinical and pathological parameters of tumor state in patients with colon and rectal cancer. Also, we identified that neoadjuvant chemotherapy/chemoradiotherapy can reverse their predictive value in more favorable way.

## 2 Materials and methods

### 2.1 Dataset analysis

The Cancer Genome Atlas (TCGA) data (TCGA-COAD and TCGA-READ datasets) and NCBI GEO (GSE190826 dataset) were used to examine the expression of SPP1, S100A4 and SPARC and to perform survival analysis in colorectal cancer patients. TCGA data included information about SPP1, S100A4 and SPARC expression, that was evaluated in the following groups of patients: a) with colorectal cancer (common group) (N=417), b) with colon cancer, including transverse colon, ascending colon, descending colon, sigmoid colon, cecum, hepatic flexure, splenic flexure (n=305), c) with rectal cancer, including rectosigmoid junction and rectum (N=112), with available clinical information and records on recurrence and survival rates (in details in **Supplementary Table S1**). Patients with advanced stage IV were excluded. GSE190826 dataset included 92 patients with rectal cancer treated with neoadjuvant chemoradiotherapy (NCRT); information about pre-treatment levels of SPP1, S100A4 and SPARC mRNA expression was obtained. The TCGA biolinks was used for retrieving RNA-seq data from the GDC database. The raw sequencing reads were processed *via* the DESeq2 R package. The raw counts were depth normalized and variance stabilized *via* the variance stabilizing transformation (VST) for downstream survival analysis.

### 2.2 CIBERSORT analysis

Cell type deconvolution of colorectal cancer TCGA RNA-seq data was performed *via* the TIMER2.0 (14) web platform, which provides a facility for robust estimation of immune infiltration levels of user-provided tumor profiles. TIMER2.0 utilizes the immunedeconv (15), an R package which integrates six distinct cell-type deconvolution algorithms, including CIBERSORT (16). CIBERSORT uses highly robust-to-noise linear support vector regression (SVR) to deconvolve the mixture of cell types of interest. Inferred immune cell-types were used to assess cell-type association of SPP1, S100A4, and SPARC genes by the Spearman correlation.

### 2.3 Clinical material

The IHC study included patients with colorectal adenocarcinoma with morphologically verified diagnosis, treated in the Department of abdominal oncology, Cancer Research Institute of Tomsk National Research Medical Center (Tomsk, Russia). The study was carried out according to Declaration of Helsinki (from 1964, revised in 1975 and 1983) and was approved by the local committee of Medical Ethics of Tomsk Cancer Research Institute; all patients signed informed consent for the study. Patients were divided as we did for TCGA cohort with the exception that the number of patients in SPARC/SPP1 group differed from S100A4 group: a) with colorectal cancer (common group for SPARC/SPP1) (N=118), b) with colon cancer (N=54), c) with rectal cancer (N=64) (**Supplementary Table S2**). For S100A4 group: a) with colorectal cancer (common group) (N=197), b) with colon cancer (N=89), c) with rectal cancer (N=107) (**Supplementary Table S3**). Patients with rectal cancer and cancer of the rectosigmoid junction received neoadjuvant chemotherapy (NAC) or chemoradiotherapy (NCRT). Five-grade Mandard Tumor Regression Grading (TRG) system was used for assessment of response in patients, where TRG1 – no residual cancer, TRG2 – residual isolated cancer cells, TRG3 – fibrosis outgrowing residual cancer, TRG4 – residual cancer outgrowing fibrosis, TRG5 – absence of regressive changes (14). All patients underwent surgical treatment. In adjuvant regime, according to indications, patients received chemotherapy under the same schemes for up to 6 months. Cases of stage IV disease were excluded.

### 2.4 Immunohistochemical analysis

FFPE tissue sections were obtained from all CRC patients after tumor resection. Immunohistochemical analysis (IHC) was carried out by standard method. Following antibodies were used: polyclonal rabbit anti-S100A4 (1:1000, PA5-82322, Thermo Fisher Scientific, USA), polyclonal goat anti-SPARC (1:80, AF941, R&D Systems, USA), polyclonal goat anti-SPP1 (1:80, AF1433, R&D Systems, USA). To visualize the antigen-antibody reaction, rabbit anti-goat IgG (1:250, VB2932894, Invitrogen, USA) or poly-HRP anti-mouse/rabbit system (Bond oracle IHC system, TA9145, Leica Biosystems, Germany) were used. The nuclei were counterstained with hematoxylin.

### 2.5 Digital quantification

Tumor tissue slides were scanned by using the Leica Aperio AT2 histoscanning station (Leica, Germany) and ScanScope software (Aperio ScanScope XT Leica). QuPath software (free from https://qupath.github.io) was used to analyze and quantify marker expression. Individual tumor regions were selected and analyzed using cell detection and cell intensity classification. “Cell: DAB OD mean” was used for the analysis of both membranous and cytoplasmic staining of SPP1, S100A4 and SPARC. Intensity thresholds were set to further subclassify cells as being negative, weak (1+), moderate (2+) or strongly positive (3+) for marker staining based upon mean nuclear DAB optical density. The results were obtained in two scales: percentage of positive cells among all counted cells per section (%) and H-score – a parameter that takes into account both the percentage of positive cells and the intensity of staining. These parameters were counted automatically by the program.

### 2.6 Immunofluorescence and confocal microscopy

For IF double staining mouse anti-CD68 monoclonal antibody (1:100, #NBP2-44539, clone KP1, Novus Biologicals); polyclonal rabbit anti-S100A4 (1:1000, PA5-82322, Thermo Fisher Scientific, USA), polyclonal goat anti-SPARC (1:80, AF941, R&D Systems, USA) and polyclonal goat anti-SPP1 (1:80, AF1433, R&D Systems, USA) were used. Combination of secondary antibodies were applied: donkey Cy3-conjugated anti-rabbit antibody (#711-165-152, Dianova, Germany, dilution 1:400), donkey AlexaFluor488-conjugated anti-mouse antibody (#715-545-150, Dianova, Germany, dilution 1:400) and donkey Cy3-conjugated anti-goat antibody (#706-167-003, Dianova, Germany, dilution 1:400). Samples were mounted with Fluoroshield Mounting Medium with DAPI (#ab104135, Abcam, USA) and analyzed by confocal microscopy. Confocal laser scanning microscopy was performed with Carl Zeiss LSM 780 NLO laser scanning spectral confocal microscope (Carl Zeiss, Germany), equipped with 40x objective. Data were acquired and analyzed with Black Zen software (RRID : SCR_018163). All three-color images were acquired using a sequential scan mode.

### 2.7 NGS-GeoMx Digital Spatial Profiler (DSP) analysis

NanoString GeoMx digital spatial profiling (DSP) was applied to perform spatially resolved RNA profiling analysis in colorectal cancer tissue. The Cancer Transcriptome Atlas (CTA) panel was used. The 97 areas of illumination (AOIs) across all slides in mixed stroma/tumor regions was selected. The resulted libraries were sequenced by the Illumina NextSeq 500 platform using 2 x 27 base paired reads. The raw counts were processed in the NanoString’s GeoMx NGS pipeline v.2.1 where they were converted to the digital count conversion (DCC) files. The GeomxTools was used for quality control (QC) and downstream analysis of the DCC files in R (17). The adjusted p-values were calculated using the Benjamini-Hochberg correction. Differential gene expression analysis between CK+ and CD45+ regions was performed using a linear mixed model (LMM) with random slope and random intercept as recommended in the GeomxTools manual.

### 2.8 Statistical analysis

Statistical analysis was performed using STATISTICA 12.0 for Windows (STATISTICA, RRID : SCR_014213) and GraphPad Prism 8.4.2 (GraphPad Prism, RRID : SCR_002798). The Mann-Whitney U-test and t-test for independent groups were implemented. The prognostic values of SPP1, S100A4 and SPARC (area under curve (AUC), confidence interval (CI), sensitivity, specificity, and cut-off value) were determined using receiver operating characteristic (ROC) analysis. The survival rates were determined by the Kaplan–Meier method, and the log-rank test was used. Cox’s proportional-hazard model was applied for survival analysis and the hazard ratio (HR [95%CI]) evaluation. Results were presented using GraphPad Prism 8.4.2 software. Results were considered to be significant with p<0,05. Data with marginal significance (p-value >0,05 and <0,1) were also discussed.

## 3 Results

### 3.1 High S100A4 mRNA expression is a robust predictor for shorter survival rates independent of cancer type

S100A4 is a pro-angiogenic factor belonging to the family of calcium-binding proteins (13). In the pathogenesis of cancer, increased S100A4 expression correlates with a high incidence of metastasis and poor prognosis in cancer (18).

To reveal the prognostic value of S100A4 mRNA expression, we performed survival analysis using TCGA data. The mRNA expression was defined further as log-normalized counts. We analyzed a combined group of CRC patients and two separate cohorts of patients with colon cancer and rectal cancer.

**Figure 1:**
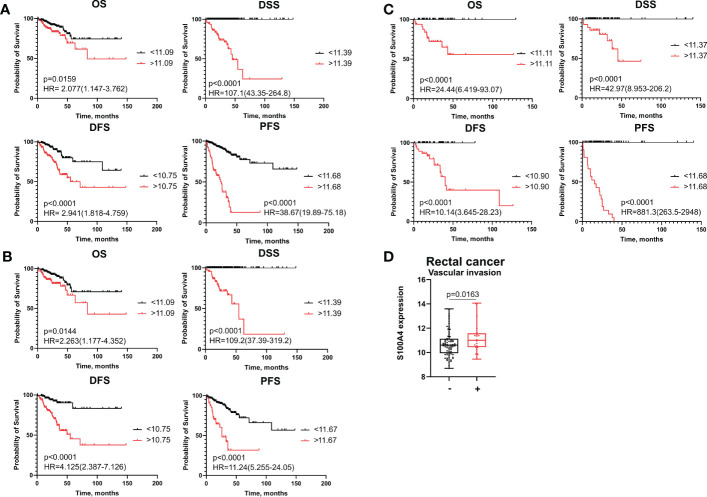
S100A4 mRNA expression is an unfavorable prognostic factor for colon and rectal cancer patients. **(A)** Kaplan–Meier curve of TCGA survival data (OS, DSS, DFS, and PFS) for high-risk and low-risk patients in combined CRC group. **(B)** Kaplan–Meier curve of TCGA survival data for high-risk and low-risk groups in colon cancer patients. **(C)** Kaplan–Meier curve of TCGA survival data for high-risk and low-risk groups of rectal cancer patients. Log-rank test p-values are shown in Kaplan–Meier curves. **(D)** S100A4 mRNA expression is associated with vascular invasion in rectal cancer patients (TCGA-READ data). Mann-Whitney U test was applied. Box plot depicts gene expression (min, Q1, median, Q3, max).

We applied Cox regression analysis, ROC analysis, and Kaplan–Meier curves to evaluate significance of S100A4 on disease prognosis in the combined CRC group, as well as CC and RC patients. In each group, all patients were categorized into high- and low-risk groups based on S100A4 expression according to the cut-off meanings determined by ROC analysis that allowed to predict overall death, death from the disease, recurrence and progression (**Supplementary Table S4**). Kaplan−Meier survival curve indicated that CRC patients in S100A4 high-risk group (>cut-off) had shorter overall survival (OS) (p=0,0159), disease-specific survival (DSS) (p<0,0001), disease-free survival (DFS) (p<0,0001), and progression-free survival (PFS) (p<0,0001) than those in low-risk group (<cut-off) (**Figure 1A**). The similar results were found for OS, DSS, DFS, and PFS in both patients with colon and rectal cancer (**Figures 1B, C**), indicating that prognostic significance of S100A4 mRNA expression does not depend on cancer type.

To investigate further, whether S100A4 mRNA expression could serve as an independent prognostic criterion of death or recurrence/progression, uni- and multivariate Cox regression analyses were applied. Univariate Cox regression analysis showed that S100A4 mRNA expression more than cut-off has prognostic value for poorer OS (HR=2,19; 95% CI [1,277-3,753], p=0,004), poorer DFS (HR=2,74; 95% CI [1,604-4,689], p=0,0002) and poorer PFS (HR=8,01; 95% CI [5,066-12,680], p<0,0001) in common CRC group (**Table 1** and **Supplementary Table S5**). In univariate COX regression high S100A4 gene expression was prognostic for worse OS (HR=2,43; 95% CI [1,331-4,438], p=0,003), worse DFS (HR=4,73; 95% CI [2,332-9,626], p<0,0001), and worse PFS (HR=4,35; 95% CI [2,521-7,524], p<0,0001) in colon cancer patients (**Table 2** and **Supplementary Table S6**). After adjusting for the clinical and pathological parameters such as age, tumor stage, tumor size, lymphovascular invasion, vascular invasion and lymph node metastasis the S100A4 mRNA expression remained an independent prognostic factor for DFS in the CRC group (HR=3,73; 95% CI [1,917-7,274], p<0,0001) and the CC group (HR=10,52; 95% CI [3,655-30,310], p<0,0001) (**Tables 1**, **2**). Multivariate Cox analysis displayed that S100A4 mRNA expression more than 11,68 (HR=9,97; 95% CI [5,800-17,150], p<0,0001), large tumor size (HR=2,41; 95% CI [1,216-4,790], p=0,011) and positive vascular invasion (HR=2,36; 95% CI [1,357-4,097], p=0,002) were associated with worse PFS in CRC patients (**Supplementary Table S5**). The same factors were indicative for the prognosis of poor PFS in colon cancer group: S100A4 mRNA expression more than 11,68 (HR=5,85; 95% CI [3,071-11,150], p<0,0001), large tumor size (HR=2,25; 95% CI [1,024-4,970], p=0,043) and positive vascular invasion (HR=2,36; 95% CI [1,252-4,460], p=0,007) (**Supplementary Table S6**). In rectal cancer patients, COX analysis did not show the prognostic significance of S100A4 mRNA expression.

**Table 1:** The prognostic significance of S100A4 mRNA levels for disease-free survival in patients with CRC revealed by univariate and multivariate COX analysis.

Parameter	Univariate			Multivariate		
	HR	95% CI	p-value	HR	95% CI	p-value
Disease-free survival						
Age *<70 years>70 years*	1,100	0,682-1,796	0,678	1,140	0,598-2,197	0,680
Stage *Early (1-2) Advanced (3)*	2,100	1,293-3,416	0,002	3,760	0,409-34,586	0,240
Tumor size *T1-2 T3-4*	2,220	1,102-4,502	0,020	1,360	0,588-3,162	0,469
Vascular invasion *Negative Positive*	1,470	0,825-2,622	0,190	1,040	0,508-2,168	0,895
Lymphovascular invasion *Negative Positive*	1,650	0,997-2,732	0,050	1,630	0,802-3,336	0,175
Lymphatic metastasis *Negative Positive*	2,220	1,382-3,595	0,001	6,440	0,747-55,591	0,090
S100A4 expression *<10,75>10,75*	2,740	1,604-4,689	0,0002	3,730	1,917-7,274	0,0001

**Table 2:** The prognostic significance of S100A4 mRNA levels for disease-free survival with colon cancer revealed by univariate and multivariate COX analysis.

Parameter	Univariate			Multivariate		
	HR	95% CI	p-value	HR	95% CI	p-value
Disease-free survival						
Age *<70 years>70 years*	1,300	0,754-2,241	0,344	1,267	0,607-1,439	0,527
Stage *Early (1-2) Advanced (3)*	1,820	1,040-3,185	0,035	0,393	0,038-4,047	0,433
Tumor size *T1-2 T3-4*	2,190	0,895-4,867	0,054	1,762	0,654-4,749	0,262
Vascular invasion *Negative Positive*	1,680	0,896-3,151	0,105	1,195	0,506-2,821	0,684
Lymphovascular invasion *Negative Positive*	1,790	1,000-3,206	0,049	1,609	0,673-3,843	0,284
Lymphatic metastasis *Negative Positive*	1,990	1,154-3,441	0,013	3,850	0,424-34,939	0,230
S100A4 expression *<10,75 >10,75*	4,730	2,332-9,626	<0,0001	10,526	3,655-30,310	<0,0001

In colon cancer patients, ROC analysis determined the most optimal cut-off meanings for S100A4 mRNA expression to predict OS, DFS and PFS with higher sensitivity and specificity (indicated in **Supplementary Table S4**). Thus, S100A4 mRNA expression more than cut-off predicted poor OS (AUC=0,956, p<0,0001), poor DSS (AUC=0,988, p<0,0001) DFS (AUC=0,997, p<0,0001), and poor PFS (AUC=0,983, p<0,0001) (**Supplementary Table S4**). In the RC group, increased gene expression of S100A4 (>11,68) was the most robust criteria for the prognosis of short PFS with the corresponding sensitivity 100% and specificity 100% (AUC=1,0, p<0,0001) (**Figure 1C** and **Supplementary Table S4**).

Statistical analysis showed that high S100A4 expression was associated with positive vascular invasion in RC patients (11,23 ± 1,26 vs. 10,75 ± 1,07; p=0,0163) (**Figure 1D**). No significant associations were found with other clinical-pathological parameters. In CRC patients and colon cancer patients, no significant differences in S100A4 gene expression that was related to clinical-pathological parameters were found.

### 3.2 Elevated SPARC mRNA expression is an accurate independent prognostic factor for poor DFS and PFS in colon but not rectal cancer

**Figure 2:**
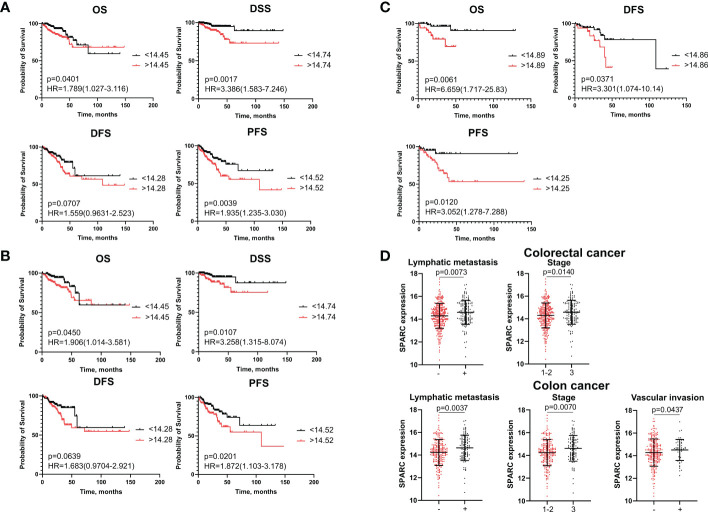
SPARC mRNA expression is associated with survival and clinical-pathological parameters. **(A)** Kaplan–Meier curve of TCGA survival data (OS, DSS, DFS, and PFS) for high-risk and low-risk patients in combined CRC group. **(B)** Kaplan–Meier curve of TCGA survival data for high-risk and low-risk groups in colon cancer patients. **(C)** Kaplan–Meier curve of TCGA survival data for high-risk and low-risk groups in rectal cancer patients. Log-rank test p-values are shown in Kaplan–Meier curves. **(D)** SPARC mRNA expression is associated with lymph node metastasis and tumor stage in CRC patients, and with lymph node metastasis, stage and vascular invasion in colon cancer patients. Student’s t-test was applied. Scatter plots depict gene expression as mean with SD.

SPARC (secreted protein acidic and rich in cysteine, also known as osteonectin or BM-40) is a calcium-binding matricellular protein. In the TME, SPARC is anti-angiogenic and affects tumor growth, extracellular matrix deposition (19). Similar to S100A4, ROC analysis allowed to divide all patients into high- and low-risk groups for prognosis of overall death, death from the disease, recurrence and progression based on SPARC mRNA level (**Supplementary Table S7**). SPARC mRNA expression more than cut-off in the high-risk group was significantly associated with worse DSS (p=0,0017) and worse PFS (p=0,0039), but with better OS (p=0,0401) in patients with CRC (**Figure 2A**). Similar tendency was shown for colon cancer patients (**Figure 2B**). In rectal cancer, SPARC mRNA expression more than cut-off was associated with poor OS (p=0,0061), poor DFS (p=0,0371) and poor PFS (p=0,0120) (**Figure 2C**). In univariate COX analysis SPARC gene expression more than cut-off predicted worse DSS (HR=2,88; 95% CI [1,377-6,006], p=0,004) and worse PFS (HR=2,06; 95% CI [1,308-3,258], p=0,002) in CRC group, as well as worse DSS (HR=2,64; 95% CI [1,141-6,110], p=0,023) and worse PFS (HR=1,98; 95% CI [1,172-3,360], p=0,010) in colon cancer patients (**Table 3** and **Supplementary Tables S8**, **S9**). After adjusting for age, tumor stage, tumor size, lymphovascular invasion, vascular invasion and lymph node metastasis, multivariate Cox regression analysis revealed that increased SPARC mRNA expression remained an independent prognostic factor for poor DSS (HR=6,65; 95% CI [2,208-20,100], p=0,0007) and poor PFS (HR=1,88; 95% CI [1,144-3,120], p=0,001) in CRC patients (**Table 3**). In colon cancer patients, SPARC mRNA expression in high-risk group also independently predicted short DSS (H=7,44; 95% CI [2,082-26,600], p=0,002) and short PFS (HR=1,83; 95% CI [1,021-3,302], p=0,042) (**Supplementary Table S9**). In RC patients, SPARC mRNA level more than cut-off was prognostic in terms of short OS (HR=5,84; 95% CI [1,404-21,421], p=0,014), short DFS (HR=2,83; 95% CI [1,019-7,907], p=0,045), and short PFS (HR=4,50; 95% CI [1,332-15,225], p=0,015) in univariate COX analysis. But it was not an independent criterion in multivariate analysis (**Supplementary Table S10**). Thus, we concluded that SPARC mRNA expression could serve as an independent prognostic factor for DFS and PFS in colon but not rectal cancer.

**Table 3:** The prognostic significance of SPARC mRNA levels in patients with CRC revealed by univariate and multivariate COX analysis.

Parameter	Univariate			Multivariate		
	HR	95% CI	p-value	HR	95% CI	p-value
Disease-specific survival						
Age *<70 years>70 years*	1,570	0,775-3,199	0,208	1,180	0,475-3,000	0,716
Stage *Early (1-2)Advanced (3)*	2,620	1,001-6,894	0,0049	5,710	0,00005-591217	0,767
Tumor size *T1-2 T3-4*	1,670	0,642-4,364	0,291	3,760	0,853-16,700	0,080
Vascular invasion *Negative Positive*	2,420	1,067-5,496	0,034	2,050	0,772-5,500	0,148
Lymphovascular invasion *Negative Positive*	1,170	0,549-2,507	0,679	1,130	0,398-3,200	0,821
Lymphatic metastasis *Negative Positive*	2,240	0,917-5,502	0,228	0,040	0-5131	0,609
SPARC expression *<14,74>14,74*	2,870	1,377-6,006	0,004	6,650	2,208-20,100	0,0007
Progression-free survival						
Age *<70 years>70 years*	1,370	0,889-2,139	0,150	1,420	0,882-2,290	0,148
Stage *Early (1-2) Advanced (3)*	0,840	0,527-1,340	0,467	6,920	0,003-1773	0,493
Tumor size *T1-2 T3-4*	1,310	0,758-2,276	0,330	1,840	0,954-3,569	0,068
Vascular invasion *Negative Positive*	1,610	0,962-2,697	0,069	1,640	0,556-2,816	0,072
Lymphovascular invasion *Negative Positive*	1,250	0,793-1978	0,333	1,480	0,844-2,600	0,170
Lymphatic metastasis *Negative Positive*	0,820	0,517-1,315	0,118	0,090	0,003-23,335	0,396
SPARC expression *<14,52>14,52*	2,060	1,308-3,258	0,002	1,880	1,144-3,120	0,001

High SPARC gene expression indicated advanced tumor stage (14,58 ± 1,06 vs. 14,3 ± 1,10; p=0,014) and positive lymph node metastasis (14,59 ± 1,05 vs. 14,29 ± 1,09; p=0,00073) in CRC (**Figure 2D**). In colon cancer patients, high SPARC mRNA expression was associated with advanced tumor stage (14,62 ± 1,13 vs. 14,24 ± 1,15; p=0,007), positive lymph node metastasis (14,64 ± 1,12 vs. 14,23 ± 1,13; p=0,003) and positive vascular invasion (14,28 ± 1,2 vs 14,51 ± 0,93, p=0,043) (**Figure 2D**). No significant differences in SPARC expression were found for clinical-pathological parameters in rectal cancer patients.

### 3.3 Increased SPP1 mRNA expression is an independent unfavorable criterion for PFS in both rectal and colon cancers

Secreted phosphoprotein 1 (SPP1, OPN, osteopontin) is an integrin-binding matricellular protein that has been found to be involved in many cellular processes such as cell signaling pathways, cell adhesion and migration, cell-mediated immunity, angiogenesis, and metastasis (20).

**Figure 3:**
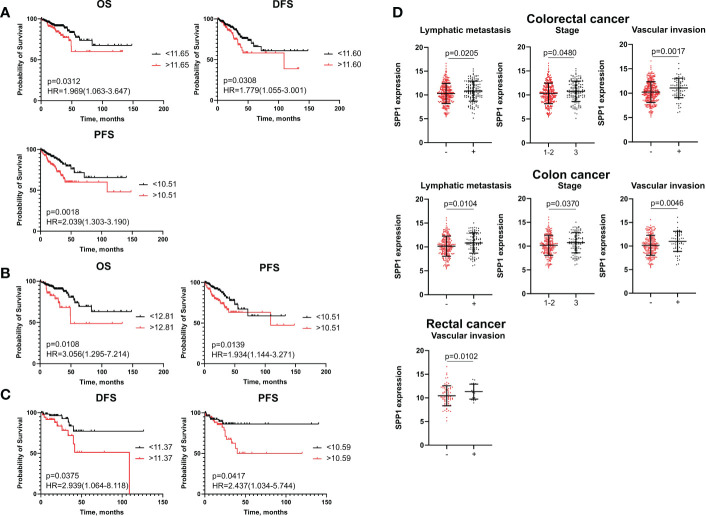
SPP1 mRNA expression is associated with poor survival and clinical-pathological parameters. **(A)** Kaplan–Meier curve of TCGA survival data (OS, DFS, and PFS) for high-risk and low-risk patients in combined CRC group. **(B)** Kaplan–Meier curve of TCGA survival data (OS and PFS) for high-risk and low-risk groups in colon cancer patients. **(C)** Kaplan–Meier curve of TCGA survival data (DFS and PFS) for high-risk and low-risk groups in rectal cancer patients. Log-rank test p-values are shown in Kaplan–Meier curves. **(D)** SPP1 mRNA expression is associated with lymph node, tumor stage and vascular invasion in CRC patients and colon cancer patients, and with vascular invasion in rectal cancer patients. Student’s t-test was applied. Scatter plots depict gene expression as mean with SD.

Kaplan–Meier analysis demonstrated that high-risk group based on SPP1 mRNA level more than cut-off had worse OS (p=0,0312), worse DFS (p=0,0308) and worse PFS (p=0,0018) compared to the low-risk group (<cut-off) in the combined CRC group (**Figure 3A**). In patients with colon cancer, OS (p=0,0108) and PFS (p=0,0139) rates were lower in cases with higher expression of SPP1 (>cut-off) (**Figure 3B**). For RC patients, the high-risk group had decreased rates of DFS (p=0,0375) and PFS (p=0,0417) (**Figure 3C**).

Univariate Cox regression analysis showed that mRNA levels of SPP1 more than cut-off were associated with decreased rates of DFS (HR=2,22; 95% CI [1,382-3,595], p=0,001) and PFS (HR=2,20; 95% CI [1,382-3,526], p=0,0009) in the CRC group (**Supplementary Table S11**). In univariate analysis, high SPP1 gene expression was prognostic for worse OS (HR=2,12; 95% CI [1,092-4,149], p=0,026) and worse PFS (HR=2,08; 95% CI [1,212-3,585], p=0,007) in colon cancer, and for worse DFS (HR=2,81; 95% CI [1,016-7,741], p=0,046) and PFS (HR=2,78; 95% CI [1,089-7,125], p=0,032) in rectal cancer (**Supplementary Tables S12**, **S13**). After including the clinical and pathological parameters in multivariate Cox analysis, the SPP1 expression more than cut-off remained independent prognostic factor for short PFS in both colon (HR=2,35; 95% CI [1,294-4,283], p=0,005) and rectal cancers (HR=3,32; 95% CI [1,124-9,809], p=0,029) patients (**Supplementary Tables S12**, **S13**).

Statistical data showed that elevated SPP1 mRNA expression correlated with positive vascular invasion (11,08 ± 2,00 vs. 10,23 ± 2,11; p=0,0017), positive lymphatic metastasis (10,82 ± 2,10 vs. 10,31 ± 2,12; p=0,0205) and advanced tumor stage (10,33 ± 2,11 vs. 10,18 ± 2,13; p=0,048) in combined CRC group. The same correlations were found in patients with colon cancer (**Figure 3D**). In the RC group, an increased SPP1 expression was related to positive vascular invasion (11,33 ± 1,58 vs. 10,44 ± 2,11; p=0,010) (**Figure 3D**).

### 3.4 S100A4, SPP1 and SPARC are expressed by tumor-associated macrophages in human colorectal cancer tissue

Using digital quantification, we performed IHC analysis of S100A4, SPP1 and SPARC in human colon and rectal cancer tissue. To increase the reproducibility and accuracy of quantification analysis, we used two methods to quantify protein expression. One of them was based on H-score, and the second included a percentage of positive cells. Correlation analysis showed that expression based on % and h-score had strong correlations (R=0,99 for S100A4; R=0,97 for SPARC, and R=0,99 for SPP1). Further, we used protein level in %, as it was more statistically significant.

**Figure 4:**
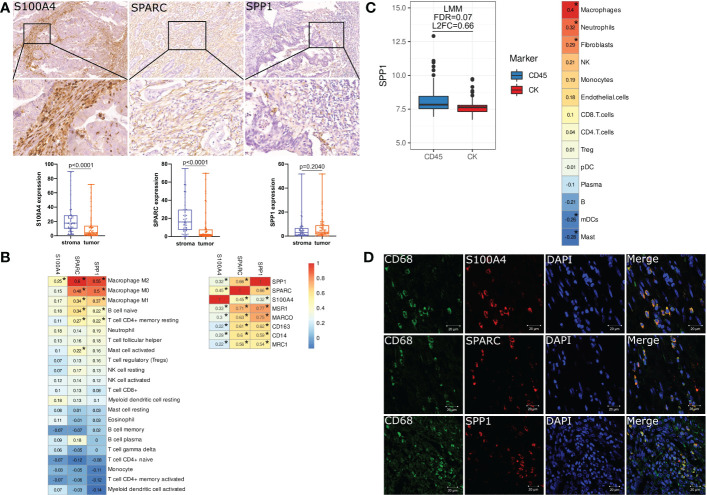
S100A4, SPARC and SPP1 are expressed by tumor-associated macrophages in colorectal cancer. **(A)** IHC representative images (x400) of stromal S100A4, SPARC and SPP1 in tumor tissue. Zoom images is given in the lower panel (x800). Stromal expression of S100A4 and SPARC is higher than tumor expression. The protein level of SPP1 does not have differences between tumor and stroma. Student’s t-test was applied. **(B)** Immune cell-type deconvolution analysis that was performed *via* the TIMER2.0 web platform using the CIBERSORT method. S100A4, SPP1 and SPARC gene expression correlates with macrophages. *: |rho|>0.2 and FDR<0.05. **(C)** Nanostring GeoMX DSP revealed increased expression of SPP1 in the immune compartment and its correlation with macrophages. *: |rho|>0.2 and FDR<0.05. **(D)** Confocal microscopy confirmed the co-localization of S100A4, SPARC and SPP1 in CD68+ TAMs. Scale bars equal 20 µm.

We demonstrated that in protein level S100A4 and SPARC are more abundantly expressed by the cells of stroma compartments compared to tumor nest [17,69 (10,03–28,65) S100A4 stroma vs. 4,18 (1,31-13,93) S100A4 tumor, p<0,0001 and 16,04 (7,29-29,62) stroma SPARC vs. 2,08 (0,78-7,72) tumor SPARC, p<0,0001)] (**Figure 4A**). SPP1 was less expressed in CRC tissue, and its expression was equal in the stromal compartment and in the tumor nest [2,93(0,63-6,67) stroma SPP1 vs. 3,31 (1,52-9,04) tumor SPP1, p=0,204) (**Figure 4A**).

Using CIBERSORT method, we demonstrated that mRNA expression of SPP1 and SPARC is significantly associated with M0, M1 and M2 macrophage phenotypes, naive B cells and CD4 T cells, while S100A4 gene expression correlates with M2 macrophage phenotype (**Figure 4B**). SPP1 and SPARC mRNA expression strongly correlated with the expression of MSR1, CD163, MRC1 and MARCO – markers associated with M2 TAM phenotype (**Figure 4B**).

Additional Nanostring analysis allowed us to reveal that SPP1 is differentially expressed in immune CD45+ and tumor cytokeratin (CK)+ regions. The gene expression of SPP1 was higher in CD45+ compartments compared to CK+ regions (FDR=0.07, L2FC=0.66). SPP1 expression in the distinct regions of CRC was mostly associated with macrophages (**Figure 4C**).

To confirm the expression of S100A4, SPP1, and SPARC in TAMs, we performed three-color IF analysis of human CRC tissue using confocal microscopy. IF analysis demonstrated that S100A4, SPP1 and SPARC were all expressed in CD68+ TAMs (**Figure 4D**).

Thus, we demonstrated that levels of S100A4, SPP1 and SPARC are mostly inherent to the stromal component, in particular macrophages. Taken into account the observations made above, we used stromal-derived protein expression of S100A4, SPP1, and SPARC for further survival and correlation analysis.

### 3.5 Stromal levels of S100A4, SPARC and SPP1 retained an unfavorable prognostic value for patient outcome but became favorable for the pathological tumor parameters

**Figure 5:**
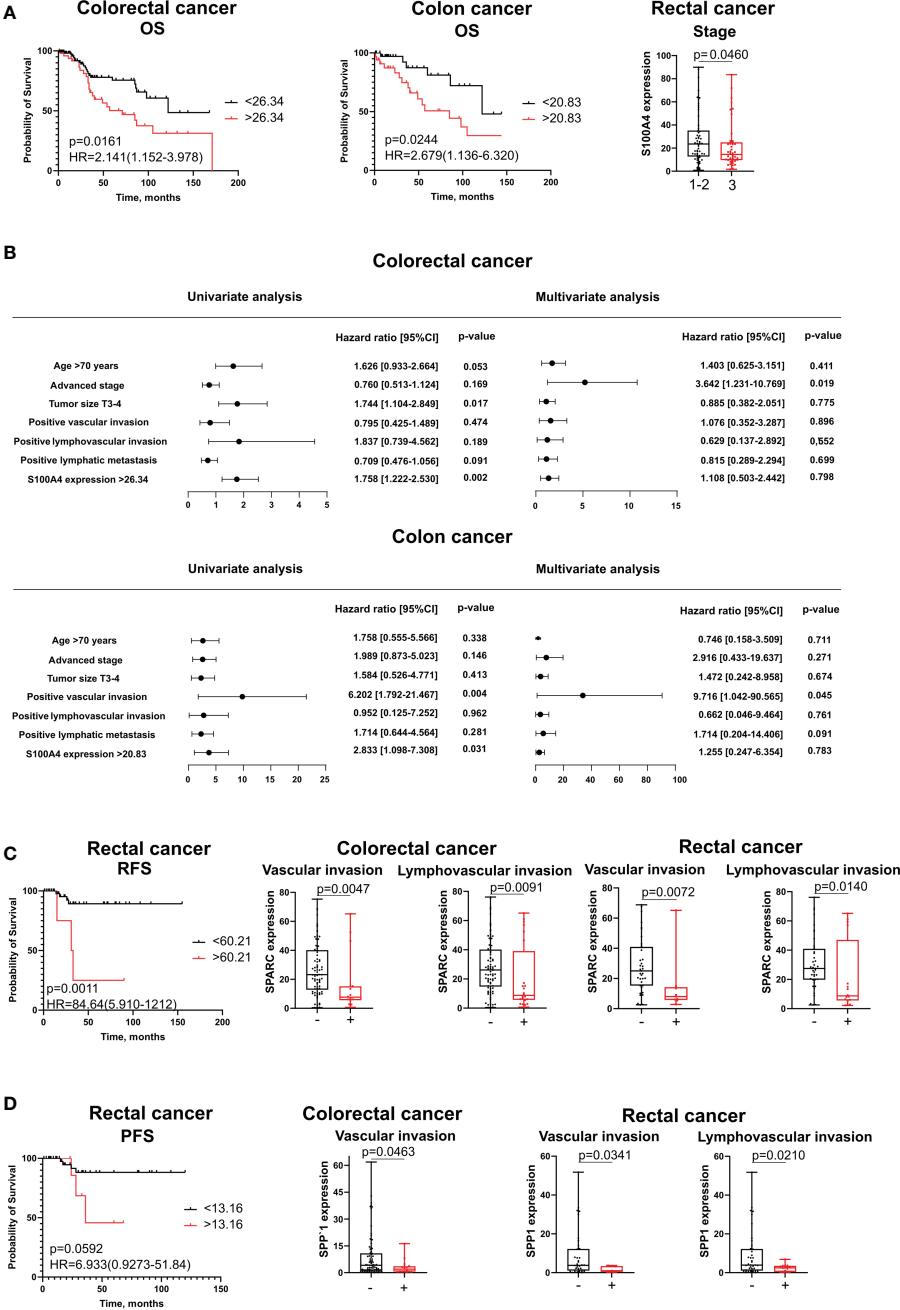
The association of stromal expression of S100A4, SPARC and SPP1 with survival and clinical-pathological parameters. **(A)** S100A4 stromal expression predicts poor OS for CRC patients and colon cancer patients, but is negatively correlated with tumor stage in rectal cancer. **(B)** The prognostic significance of S100A4 protein levels for overall survival in patients with CRC and CC revealed by univariate and multivariate COX analysis. **(C)** SPARC stromal expression is an unfavorable parameter for RFS in rectal cancer patients. SPARC protein expression is negatively associated with clinical-pathological parameters in combined CRC group and in rectal cancer patients. **(D)** SPP1 stromal expression is unfavorable for PFS in patients with rectal cancer. SPP1 protein expression is negatively associated with clinical-pathological parameters in combined CRC group and in rectal cancer patients. Log-rank test p-values are shown in Kaplan-Meier plots. Mann-Whitney U test was applied for the comparison of two groups. Box plots depict protein expression (min, Q1, median, Q3, max).

Protein levels of S100A4, SPARC and SPP1 remained unfavorable parameters for survival. In univariate COX analysis and Kaplan–Meier survival analysis, the protein level of S100A4 in high-risk group (expression more than cut-off meaning) remained prognostic only for OS in both combined CRC group of patients (HR=2,141; 95% CI [1,152-3,978], p=0,016) and patients with colon cancer (HR=2,679; 95% CI [1,136-6,320], p=0,024) (**Figures 5A, B**). In multivariate Cox analysis, S100A4 mRNA expression was not an independent parameter. High protein expression of SPARC and SPP1 was associated with shorter recurrence-free survival (RFS) and PFS, respectively, in patients with rectal cancer (**Figures 5C, D**, respectively). However, these parameters were not prognostic according to univariate and multivariate Cox analysis.

Interesting that in contrast to mRNA expression, increased protein expression of S100A4, SPARC and SPP1 was a favorable criterion for clinical and pathological parameters. Thus, S100A4 expression was higher in rectal cancer patients having tumor stages I-II compared to patients with stage III (28,20 ± 22,29 vs 21,47 ± 19,29, p=0,046) (**Figure 5A**). SPARC expression was lower in patients with positive lymphovascular invasion (8,76 (5,62;39,13) vs. 26,19 (14,74;40,15), p=0,0091) and positive vascular invasion (7,68 (5,62;15,19) vs. 23,26 (12,84;40,15), p=0,0047) in CRC group; similar trend was observed in the same groups of RC patients (8,76 (5,62;47,06) vs. 27,53 (19,84;41,00), p=0,014; 8,04 (5,78;14,22) vs. 25,06 (15,30;41,00), p=0,0072) (**Figure 5C**). Low protein expression of SPP1 was associated with positive vascular invasion in CRC (1,98 (0,60;3,62) vs. 4,14 (1,23;10,80), p=0,0463) and RC (1,01 (0,41;3,43) vs. 3,75 (1,08;12,23), p=0,0341) and with positive lymphovascular invasion in RC patients (2,47 (0,42;3,53) vs. 3,92 (1,08;12,23), p=0,0210) (**Figure 5D**). No significant associations were found for S100A4, SPARC, and SPP1 with other clinical and pathological parameters.

### 3.6 Neoadjuvant chemotherapy/chemoradiotherapy reverse the prognostic value of S100A4

**Figure 6:**
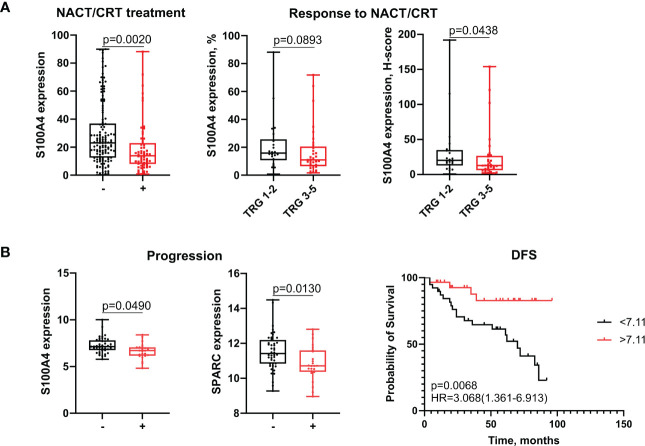
S100A4 is associated with improved outcomes in patients undergone neoadjuvant chemotherapy-based treatment. **(A)** Stromal expression of S100A4 decreased in treated patients and correlated with favorable response to NACT/CRT. **(B)** S100A4 and SPARC mRNA expression decreased in non-responders with progression. S100A4 high-risk score predicts better DFS. Mann-Whitney U test was applied. Box plots depict protein **(A)** and gene **(B)** expression (min, Q1, median, Q3, max). Log-rank test p-value is shown in Kaplan-Meier plot.

Finally, we found that neoadjuvant chemotherapy (NACT)/chemoradiotherapy (NCRT) can reverse the activity of S100A4 from the pro-tumor to favorable one. In general, it can be hypothesized, that pro-angiogenic factors can induce formation of blood vessels with different functionality – with different permeability for soluble factors or infiltration of immune cells from one side, and different permeability for cancer cells enhancing metastasis from another side (21, 22). It can be assumed that specific type of vasculature can be beneficial for tumor growth before the treatment onset, while the same type of vasculature can be converted to the favorable for patients once chemotherapy is applied (23, 24). The possible explanation of such effect can be enhanced permeability for the chemotherapy agent or for the selective anti-tumor immune cells. The idea of this study was based on our recent observation that chemotherapy can induce reprogramming of TAMs and launch the dysregulation in the expression of angiogenesis-associated factors (data not shown). Here, we found that stromal levels of S100A4 after treatment were lower in patients treated with NACT/NCRT compared to untreated patients (29,24 ± 22,49 vs. 19,04 ± 17,97, p=0,002), indicating that chemotherapy-based treatment can suppress its expression (**Figure 6A**). Surprisingly, post-treatment stromal expression of S100A4 was higher in patients who have better response to NACT/NCRT (20,17 (12,87,35,00) for TRG1-2 vs. 12,78 (6,31;26,52) for TRG3-5, p=0,0438) (**Figure 6A**). Response to NACT/NCRT was estimated by Mandard Tumor Regression Grading (TRG) system.

Using GSE190826 cohort we showed that pre-treatment S100A4 mRNA expression in rectal cancer patients who had not achieve pathological complete response (pCR) after CRT and suffered from the recurrence, was lower than in patients without progression (7,25 ± 0,81 vs. 6,64 ± 0,82, p=0,049) (**Figure 6B**). The same association was found for SPARC (11,52 ± 1,06 vs. 10,85 ± 0,98, p=0,013) (**Figure 6B**). Moreover, among non-pCR patients, DFS survival was better in cases with higher expression of S100A4 compared to lower expression (HR=3,068; 95% CI [1,361-6,913], p=0,0068) (**Figure 6B**).

Our previous observations indicated that S100A4 can be expressed by both M1 and M2 macrophages in tumor tissues (unpublished data). We suppose that chemotherapy can induce re-population of S100A4-expressed M1 and M2 macrophages in tumors.

## 4 Discussion

Currently, CRC prognosis is largely based on clinical and pathological parameters and focuses on the cancer stage at the time of diagnosis (25). Clinically validated prognostic biomarkers that can identify “high-risk” CRC patients are currently missing (25). Among cells of immune infiltrate, only T lymphocytes were included in Immunoscore classifier that was proposed for survival prediction: patients with “hot” tumors (where CD3+ and CD8+ T cells were detected) exhibit better RFS than patients with “cold” tumors (26, 27).

Here, for the first time, we performed the complex analysis of mRNA expression using TCGA and GEO datasets, and protein expression using quantitative IHC of clinical samples for crucial regulators of tumor angiogenesis and tumor progression, expressed by tumor-associated macrophages: S100A4, SPP1 and SPARC (13). We considered colon and rectal cancer as two tumor entities as accumulating clinical data showed their differences in the prognosis and treatment strategies (28).

Using TCGA, survival analysis demonstrated that S100A4, SPP1 and SPARC could be promising predictors for the unfavorable outcome. In particular, S100A4 accurately predicted poor survival with high sensitivity and specificity for patients with CRC independently on cancer type, so S100A4 can be a universal unfavorable predictor in colon and rectal cancers. SPARC mRNA expression was a more specific marker for colon cancer patients. SPP1 mRNA expression was found to have a more significant predictive value for PFS in both rectal and colon cancers. Multivariate COX analysis also revealed that prognostic model consisting of S100A4 (HR=8,43; 95% CI [5,296-13,426], p<0,0001), SPARC (HR=1,86; 95% CI [1,141-3,041], p=0,012) and SPP1 (HR=1,86; 95% CI [1,124-308], p=0,058) can be established for the PFS in CRC cohort. Literature data indicate that in several cohorts of CRC, high SPP1 gene mRNA expression was associated with shorter OS, higher S100A4 expression – with shorter DFS and OS, and high SPARС expression – with worse DFS (29–33). These cohorts include patients with I-IV stages of CRC or only colon cancer, or only rectal cancer. We for the first time performed complex analysis of S100A4, SPP1 and SPARC mRNA levels in terms of survival rates in common CRC groups and in patients with colon and rectal cancer separately.

Next, we found significant correlation of S100A4, SPP1 and SPARC with the amount and M2 phenotype of TAMs. Confocal analysis confirmed the co-expression of these proteins in CD68+ TAMs in human CRC tissue. The co-expression of S100A4 and SPARC in CD68+ TAMs in human CRC tissue was demonstrated by us for the first time. We were able to find only one study describing the co-localization of CD68 and SPP1 in tumor stromal components in human CRC (34). Additionally, Nanostring technology allowed to find elevated expression of SPP1 in CD45+ immune compartment compared to CK+ tumor cell compartment as well as strong correlation of SPP1 with macrophage count in CRC tissues.

We found that, in contrast to mRNA expression level, S100A4, SPP1 and SPARC, stromal level negatively correlated with clinical-pathological parameters. These data correspond to the results found for other cohorts analyzed by IHC using tissue microarrays. In a cohort of 134 patients with CRC, negative correlations were found between SPP1 expression and distant metastasis, tumor invasion, tumor grade, and recurrence (35). In a cohort of 114 patients with colon cancer, a significant negative association was observed for SPARC expression in mesenchymal and stromal cells with the differentiation of tumors (36). For S100A4, several studies did not show any association of its protein expression with pathological parameters other than survival rates (32, 37, 38).

Interestingly, that stromal expression of SPARC and SPP1 was prognostic for the reduced RFS and PFS, respectively, only in rectal cancer patients, while S100A4 correlated with poor OS rates in CRC and colon cancer patients. Such controversial data can be explained by the dual role of TAMs in CRC progression. A few reports indicate that high amounts of TAMs that are the most abundant innate immune cell population in CRC are beneficial to CRC patients (39, 40).

Finally, we found that neoadjuvant chemotherapy (NACT)/chemoradiotherapy (NCRT) can reverse the association of S100A4 with prognosis from the tumor progression-associated to favorable one. Accumulating evidence showed that chemotherapy can induce re-polarization of macrophages in the TME (8). Chemotherapy-based treatment suppressed stromal expression of S100A4. Notably, S100A4 stromal levels were higher in patients with better response to NACT/CRT, and S100A4 mRNA levels predicted better DFS among non-responders. It can be explained by the mechanism of re-population of TAMs after chemotherapeutic intervention. It can be also assumed that specific type of vasculature can be beneficial for tumor growth before the treatment onset, while the same type of vasculature can be converted to the favorable for patients once chemotherapy is applied (23, 24).

Thus, we demonstrated high prognostic significance of angiogenesis-associated factors S100A4, SPP1 and SPARC that are produced by TAMs in colorectal cancer. Our findings can help improve the prognosis of patients with CRC based on S100A4, SPP1 and SPARC expression levels. S100A4, SPP1 and SPARC can be helpful targets for developing novel immunotherapy and anti-angiogenic therapy approaches.

## Data availability statement

All data needed to evaluate the conclusions in the paper are presented in the paper and the **Supplementary Material**. Additional data related to this paper may be requested from authors. GEO data set was accessed *via* NCBI GEO Repositorium: https://www.ncbi.nlm.nih.gov/geo/query/acc.cgi?acc=GSE190826. TCGA-COAD and TCGA-READ data sets were obtained from NIH GDC data portal: https://portal.gdc.cancer.gov/. Results of NGS-GeoMx DSP analysis are presented in NCBI data portal (GSE221924): https://www.ncbi.nlm.nih.gov/geo/query/acc.cgi?acc=GSE221924.

## Ethics statement 

This study was performed in line with the principles of the Declaration of Helsinki. Approval was granted by the local committee of Medical Ethics of Tomsk Cancer Research Institute. Informed consent was obtained from all individual participants included in the study.

## Author contributions

The study was conceptualized by IL and JK; EK, MR, TS, LT performed experiments and analyzed data; PI prepared the bioinformatics data analysis; AT, SA, AD, LZ and NC provided clinical data and enrolled the patients; IL, EK and MR wrote the manuscript with input from all authors. IL and JK interpreted data and contributed to the discussion.
