# Supplementary material for: Angiogenesis regulators S100A4, SPARC and SPP1 correlate with macrophage infiltration and are prognostic biomarkers in colon and rectal cancers
Source: Front Oncol. 2023 Feb 21;13:1058337. doi: 10.3389/fonc.2023.1058337 (PMC9989292; doi:10.3389/fonc.2023.1058337)
Supplement: Supplementary file 1 [file DataSheet_1.docx]

**Supplementary Table S1 – Clinical and pathological parameters of colorectal cancer, colon cancer and rectal cancer patients obtained from TCGA database**

| TCGA data clinical & pathological parameters | Colorectal cancer  N=417 | Colon cancer  N=305 | Rectal cancer  N=112 |
| --- | --- | --- | --- |
| Mean age | 66,4±12,7 | 67,2±13 | 64,2±11,6 |
| Sex  Male  Female | N=217 (52%)  N=200 (48%) | N=160 (53%)  N=145 (47%) | N=57 (51%)  N=55 (49%) |
| Stage  1  2  3  No data | N=92 (22%)  N=179 (43%)  N=136 (33%)  N=10 (2%) | N=64 (21%)  N=137 (45%)  N=97 (32%)  N=7 (2%) | N=28 (25%)  N=42 (37%)  N=39 (35%)  N=3 (3%) |
| T1  T2  T3  T4 | N=17 (4%)  N=89 (21%)  N=281 (68%)  N=30 (7%) | N=9 (3%)  N=67 (22%)  N=205 (67%)  N=24 (8%) | N=8 (7%)  N=22 (20%)  N=76 (68%)  N=6 (5%) |
| Lymph node metastasis  Yes  No | N=140 (33%)  N=277 (67%) | N=99 (32%)  N=206 (68%) | N=41 (36%)  N=71 (64%) |
| Lymphovascular invasion  Yes  No  No data | N=140 (33%)  N=243 (59%)  N=34 (8%) | N=99 (32%)  N=179 (59%)  N=27 (9%) | N=41 (36%)  N=64 (57%)  N=7 (7%) |
| Vascular invasion  Yes  No  No data | N=76 (18%)  N=293 (71%)  N=48 (11%) | N=56 (18%)  N=215 (71%)  N=34 (11%) | N=20 (18%)  N=78 (70%)  N=14 (12%) |
| Progression  Yes  No  No data | N=80 (19%)  N=338 (81%)  N=0 (0%) | N=58 (19%)  N=247 (81%)  N=0 (0%) | N=22 (20%)  N=90 (80%)  N=0 (0%) |

**Supplementary Table S2 – Clinical and pathological parameters of colorectal cancer, colon cancer and rectal cancer patients from IHC study for SPARC and SPP1 markers**

| IHC group data clinical & pathological parameters for SPARC & SPP1 | Colorectal cancer  N=118 | Colon cancer  N=54 | Rectal cancer  N=64 |
| --- | --- | --- | --- |
| Mean age | 66,3±10,3 | 67,4±9,7 | 65,6±10,7 |
| Sex  Male  Female | N=63 (53%)  N=55 (47%) | N=26 (48%)  N=28 (52%) | N=38 (60%)  N=26 (40%) |
| Stage  1  2  3  No data | N=17 (14%)  N=48 (41%)  N=43 (37%)  N=10 (8%) | N=7 (13%)  N=22 (41%)  N=19 (35%)  N=6 (11%) | N=10 (15%)  N=26 (41%)  N=24 (38%)  N=4 (6%) |
| T1  T2  T3  T4  No data | N=2 (2%)  N=27 (22%)  N=43 (37%)  N=37 (31%)  N=9 (8%) | N=1 (2%)  N=12 (22%)  N=13 (24%)  N=22 (41%)  N=6 (11%) | N=1 (2%)  N=15 (23%)  N=30 (47%)  N=15 (23%)  N=3 (5%) |
| Lymph node metastasis  Yes  No  No data | N=38 (32%)  N=71 (60%)  N=9 (8%) | N=15 (28%)  N=33 (61%)  N=6 (11%) | N=23 (36%)  N=38 (59%)  N=3 (5%) |
| Lymphovascular invasion  Yes  No  No data | N=24 (20%)  N=82 (70%)  N=12 (10%) | N=8 (15%)  N=41 (76%)  N=5 (9%) | N=16 (25%)  N=41 (64%)  N=7 (11%) |
| Vascular invasion  Yes  No  No data | N=16 (14%)  N=78 (66%)  N=24 (20%) | N=7 (13%)  N=41 (76%)  N=6 (11%) | N=9 (14%)  N=37 (58%)  N=18 (28%) |
| Hematogenous metastasis  Yes  No | N=11 (9%)  N=107 (91%) | N=4 (7%)  N=50 (93%) | N=7 (11%)  N=57 (89%) |
| Recurrence  Yes  No | N=9 (8%)  N=109 (92%) | N=1 (2%)  N=53 (98%) | N=8 (12%)  N=56 (88%) |
| NACT/NCRT  Yes  No | N=32 (27%)  N=86 (73%) | Non treated | N=34 (53%)  N=30 (47%) |
| NACT/NCRT Response  TRG1-2  TRG3-5 | -  - | Non treated | N=13 (41%)  N=19 (59%) |

Notes: NACT/NCRT – neoadjuvant chemotherapy/ neoadjuvant chemoradiotherapy

**Supplementary Table S3 – Clinical and pathological parameters of colorectal cancer, colon cancer and rectal cancer patients from IHC study for S100A4 marker**

| IHC group data clinical & pathological parameters for S100A4 | Colorectal cancer  N=197 | Colon cancer  N=89 | Rectal cancer  N=107 |
| --- | --- | --- | --- |
| Mean age | 65,5±10,4 | 67,9±10,4 | 63,9±10,1 |
| Sex  Male  Female | N=111 (56%)  N=86 (44%) | N=45 (51%)  N=44 (49%) | N=65 (61%)  N=42 (39%) |
| Stage  1  2  3  No data | N=29 (15%)  N=85 (43%)  N=73 (37%)  N=10 (5%) | N=15 (17%)  N=41 (46%)  N=27 (30%)  N=6 (7%) | N=14 (13%)  N=44 (41%)  N=46 (43%)  N=3 (3%) |
| T1  T2  T3  T4  No data | N=2 (1%)  N=40 (20%)  N=73 (37%)  N=72 (36%)  N=10 (6%) | N=1 (1%)  N=20 (22%)  N=25 (28%)  N=37 (42%)  N=6 (7%) | N=1 (1%)  N=20 (19%)  N=48 (45%)  N=35 (32%)  N=3 (3%) |
| Lymph node metastasis  Yes  No  No data | N=64 (33%)  N=121 (62%)  N=11 (5%) | N=24 (27%)  N=58 (65%)  N=7 (8%) | N=40 (38%)  N=63 (59%)  N=3 (3%) |
| Lymphovascular invasion  Yes  No  No data | N=39 (78%)  N=153 (20%)  N=5 (2%) | N=14 (16%)  N=73 (82%)  N=2 (2%) | N=80 (75%)  N=24 (22%)  N=3 (3%) |
| Vascular invasion  Yes  No  No data | N=24 (12%)  N=155 (79%)  N=18 (9%) | N=9 (10%)  N=76 (85%)  N=4 (5%) | N=14 (13%)  N=79 (74%)  N=14 (13%) |
| Hematogenous metastasis  Yes  No | N=19 (10%)  N=178 (90%) | N=7 (8%)  N=82 (92%) | N=12 (11%)  N=95 (89%) |
| Recurrence  Yes  No | N=14 (7%)  N=183 (93%) | N=3 (3%)  N=86 (97%) | N=11 (10%)  N=96 (90%) |
| NACT/NCRT  Yes  No | N=60 (31%)  N=137 (69%) | Non treated | N=55 (52%)  N=52 (48%) |
| NACT/NCRT Response  TRG1-2  TRG3-5 | -  - | Non treated | N=22 (40%)  N=33 (60%) |

Notes: NACT/NCRT – neoadjuvant chemotherapy/ neoadjuvant chemoradiotherapy

**Supplementary Table S4. Parameters of ROC analysis defined an optimal cut-off value for S100A4 mRNA expression**

| **Parameters** | **CRC** | **CC** | **RC** |
| --- | --- | --- | --- |
| **Overall survival** | AUC=0,7452; p<0,0001  Sens=100%  Spec=60,7%  (Cut-off= 11,09) | AUC=0,9558; p<0,0001  Sens=97,67%  Spec=93,89%  (Cut-off=11,09) | AUC=0,8118; p=0,0012  Sens=100%  Spec=73,53%  (Cut-off=11,11) |
| **Disease-specific survival** | AUC=0,8370; p<0,0001  Sens=100%  Spec=80,75%  (Cut-off= 11,39) | AUC=0,9884; p<0,0001  Sens=100%  Spec=92,55%  (Cut-off=11,39) | AUC=0,7885; p=0,0067  Sens=100% Spec=78,85%  (Cut-off=11,37) |
| **Disease-free survival** | AUC=0,6538; p<0,0001  Sens=67,65%  Spec=60,55%  (Cut-off= 10,75) | AUC=0,9975; p<0,0001  Sens=100% Spec=97,02%  (Cut-off=10,75) | AUC=0,7493; p=0,0015  Sens=100%  Spec=70,33%  (Cut-off=10,9) |
| **Progression-free survival** | AUC=0,9877;p<0,0001  Sens=98,77%  Spec=100%  (Cut-off= 11,68) | AUC=0,9831;p<0,0001  Sens=98,31%  Spec=100%  (Cut-off=11,67) | AUC=1,0; p<0,0001  Sens=100%  Spec=100%  (Cut-off=11,68) |

Notes: AUC – area under curve; CRC – colorectal cancer; CC – colon cancer; RC – rectal cancer; sens – sensitivity; spec – specificity.

**Supplementary Table S5. The prognostic significance of S100A4 mRNA levels in patients with CRC revealed by univariate and multivariate COX analysis**

|  | Univariate | | |  | Multivariate | | |
| --- | --- | --- | --- | --- | --- | --- | --- |
| Parameter | HR | 95% CI | P-value |  | HR | 95% CI | P-value |
| **Overall survival** |  |  |  |  |  |  |  |
| Age  *<70 years*  *>70 years* | 3,202 | 1,811-5,667 | <0,0001 |  | 3,070 | 1,591-5,936 | 0,0008 |
| Stage  *Early (1-2)*  *Advanced (3)* | 2,171 | 1,234-3,840 | 0,007 |  | 3,320 | 0,360-30,712 | 0,288 |
| Tumor size  *T1-2*  *T3-4* | 2,054 | 0,926-4,575 | 0,070 |  | 1,000 | 0,394-2,532 | 0,999 |
| Vascular invasion  *Negative*  *Positive* | 1,423 | 0,766-2,639 | 0,263 |  | 1,020 | 0,505-2,073 | 0,947 |
| Lymphovascular invasion  *Negative*  *Positive* | 1,634 | 0,930-2,868 | 0,080 |  | 1,290 | 0,613-2,744 | 0,495 |
| Lymphatic metastasis  *Negative*  *Positive* | 2,422 | 1,411-4,161 | 0,001 |  | 8,550 | 0,955-76,645 | 0,054 |
| S100A4 expression  *<11,09*  *>11,09* | 2,196 | 1,277-3,753 | 0,004 |  | 3,040 | 1,602-5,800 | 0,0006 |
| **Disease-specific survival** |  |  |  |  |  |  |  |
| Age  *<70 years*  *>70 years* | 1,570 | 0,775-3,199 | 0,208 |  | 0,620 | 0,248-1,545 | 0,545 |
| Stage  *Early (1-2)*  *Advanced (3)* | 2,620 | 1,001-6,894 | 0,0049 |  | Invalid | | 0,990 |
| Tumor size  *T1-2*  *T3-4* | 1,670 | 0,642-4,364 | 0,291 |  | 0,250 | 0,056-1,170 | 0,079 |
| Vascular invasion  *Negative*  *Positive* | 2,420 | 1,067-5,496 | 0,034 |  | 0,360 | 0,129-1,014 | 0,053 |
| Lymphovascular invasion  *Negative*  *Positive* | 1,170 | 0,549-2,507 | 0,679 |  | 1,030 | 0,376-2,856 | 0,943 |
| Lymphatic metastasis  *Negative*  *Positive* | 2,240 | 0,917-5,502 | 0,228 |  | Invalid | | 0,990 |
| S100A4 expression  *<11,39*  *>11,39* | Invalid | | 0,986 |  | Invalid | | 0,987 |
| **Progression-free survival** |  |  |  |  |  |  |  |
| Age  *<70 years*  *>70 years* | 1,370 | 0,889-2,139 | 0,150 |  | 1,350 | 0,833-2,200 | 0,219 |
| Stage  *Early (1-2)*  *Advanced (3)* | 0,840 | 0,527-1,340 | 0,467 |  | 6,430 | 0,003-13262 | 0,632 |
| Tumor size  *T1-2*  *T3-4* | 1,310 | 0,758-2,276 | 0,330 |  | 2,410 | 1,217-4,790 | 0,001 |
| Vascular invasion  *Negative*  *Positive* | 1,610 | 0,962-2,697 | 0,069 |  | 2,360 | 1,364-4,110 | 0,002 |
| Lymphovascular invasion  *Negative*  *Positive* | 1,250 | 0,793-1978 | 0,333 |  | 1,230 | 0,708-2,160 | 0,453 |
| Lymphatic metastasis  *Negative*  *Positive* | 0,820 | 0,517-1,315 | 0,118 |  | 0,090 | 0,00004-179,250 | 0,529 |
| S100A4 expression  *<11,68*  *>11,68* | 8,010 | 5,066-12,680 | <0,0001 |  | 9,970 | 5,800-17,150 | <0,0001 |

**Supplementary Table S6. The prognostic significance of S100A4 mRNA levels in patients with colon cancer revealed by univariate and multivariate COX analysis**

|  | Univariate | | |  | Multivariate | | |
| --- | --- | --- | --- | --- | --- | --- | --- |
| Parameter | HR | 95% CI | P-value |  | HR | 95% CI | P-value |
| **Overall survival** |  |  |  |  |  |  |  |
| Age  *<70 years*  *>70 years* | 2,760 | 1,458-5,234 | 0,001 |  | 2,901 | 1,408-6,006 | 0,003 |
| Stage  *Early (1-2)*  *Advanced (3)* | 2,080 | 1,119-3,877 | 0,02 |  | 0,270 | 0,027-2,681 | 0,264 |
| Tumor size  *T1-2*  *T3-4* | 2,290 | 0,899-5861 | 0,81 |  | 1,320 | 0,440-3,968 | 0,618 |
| Vascular invasion  *Negative*  *Positive* | 1,330 | 0,670-2,662 | 0,409 |  | 0,830 | 0,373-1,868 | 0,660 |
| Lymphovascular invasion  *Negative*  *Positive* | 2,210 | 1,184-4,127 | 0,0127 |  | 1,470 | 0,617-3,491 | 0,385 |
| Lymphatic metastasis  *Negative*  *Positive* | 2,390 | 1,314-4,367 | 0,004 |  | 10,270 | 1,090-96,718 | 0,041 |
| S100A4 expression  *<11,09*  *>11,09* | 2,430 | 1,331-4,438 | 0,003 |  | 2,900 | 1,453-5,822 | 0,186 |
| **Disease-specific survival** |  |  |  |  |  |  |  |
| Age  *<70 years*  *>70 years* | 1,740 | 0,745-3,988 | 0,202 |  | 1,290 | 0,736-2,288 | 0,570 |
| Stage  *Early (1-2)*  *Advanced (3)* | 0,620 | 0,231-1,703 | 0,361 |  | 3,080 | 0,001-6205,877 | 0,771 |
| Tumor size  *T1-2*  *T3-4* | 1,110 | 0,413-3,010 | 0,831 |  | 1,900 | 0,855-4,238 | 0,114 |
| Vascular invasion  *Negative*  *Positive* | 1,530 | 0,5447-4,333 | 0,415 |  | 2,390 | 1,270-4,523 | 0,006 |
| Lymphovascular invasion  *Negative*  *Positive* | 2,220 | 0,943-5,241 | 0,067 |  | 53,000 | 0,829-2,848 | 0,172 |
| Lymphatic metastasis  *Negative*  *Positive* | 0,740 | 0,291-1,878 | 0,526 |  | 0,278 | 0,000-560,041 | 0,741 |
| S100A4 expression  *<11,39*  *>11,39* | Invalid | Invalid | 0,987 |  | 3,430 | 1,886-6,242 | <0,0001 |
| **Progression-free survival** |  |  |  |  |  |  |  |
| Age  *<70 years*  *>70 years* | 1,450 | 0,867-2,449 | 0,154 |  | 1,440 | 0,817-2,540 | 0,206 |
| Stage  *Early (1-2)*  *Advanced (3)* | 0,890 | 0,514-1,550 | 0,689 |  | 9,660 | Invalid | 0,567 |
| Tumor size  *T1-2*  *T3-4* | 1,370 | 0,713-2,663 | 0,339 |  | 2,250 | 1,024-4,970 | 0,043 |
| Vascular invasion  *Negative*  *Positive* | 1,760 | 0,983-3,155 | 0,056 |  | 2,360 | 1,255-4,470 | 0,007 |
| Lymphovascular invasion  *Negative*  *Positive* | 1,180 | 0,684-2,050 | 0,544 |  | 1,250 | 0,645-2,430 | 0,507 |
| Lymphatic metastasis  *Negative*  *Positive* | 0,870 | 0,400-1,519 | 0,636 |  | 0,070 | 0,000-175,660 | 0,511 |
| S100A4 expression  *<11,68*  *>11,68* | 4,350 | 2,521-7,524 | <0,0001 |  | 5,850 | 3,071-11,150 | <0,0001 |

**Supplementary Table S7. Parameters of ROC analysis defined an optimal cut-off value for SPARC mRNA expression**

| **Parameter** | **CRC** | **CC** | **RC** |
| --- | --- | --- | --- |
| **Overall survival** | AUC=0,6145; p=0,007  Sens=62,26% Spec=51,65%  (Cut-off= 14,45) | AUC=0,6036; p=0,0295  Sens=72,09% Spec=48,09 %  (Cut-off=14,45) | AUC=0,6892; p=0,049  Sens=70%  Spec=68,63%  (Cut-off=14,89) |
| **Disease-specific survival** | AUC=0,6363; p=0,0115  Sens=64,52%  Spec=65,03%  (Cut-off= 14,74) | AUC=0,6332; p=0,033  Sens=69,57%  Spec=61,7%  (Cut-off=14,74) | Not significant |
| **Disease-free survival** | AUC=0,5756; p=0,049  Sens=67,65% Spec=47,4%  (Cut-off= 14,28) | AUC=0,5961; p=0,0152  Sens=71,01%  Spec=49,58%  (Cut-off=14,28) | Not significant |
| **Progression-free survival** | AUC=0,6307; p=0,0003  Sens=63,75%  Spec=57,14%  (Cut-off= 14,52) | AUC=0,6180; p=0,0052  Sens=68,97% Spec=49,39%  (Cut-off=14,52) | AUC=0,6606; p=0,019  Sens=86,36%  Spec=46,67%  (Cut-off=14,25) |

**Supplementary Table S8. The prognostic significance of SPARC mRNA levels in patients with CRC revealed by univariate and multivariate COX analysis**

|  | Univariate | | |  | | Multivariate | | | | |
| --- | --- | --- | --- | --- | --- | --- | --- | --- | --- | --- |
| Parameter | HR | 95% CI | P-value | |  | | HR | 95% CI | P-value |  |
| **Overall survival** |  |  |  | |  | |  |  |  |  |
| Age  *<70 years*  *>70 years* | 3,200 | 1,811-5,667 | <0,0001 | |  | | 3,580 | 1,853-6,915 | 0,0001 |  |
| Stage  *Early (1-2)*  *Advanced (3)* | 2,170 | 1,234-3,840 | 0,007 | |  | | 0,644 | 0,075-5,518 | 0,688 |  |
| Tumor size  *T1-2*  *T3-4* | 2,050 | 0,926-4,575 | 0,070 | |  | | 1,260 | 0,511-3,104 | 0,615 |  |
| Vascular invasion  *Negative*  *Positive* | 1,420 | 0,766-2,639 | 0,263 | |  | | 1,250 | 0,622-2,514 | 0,528 |  |
| Lymphovascular invasion  *Negative*  *Positive* | 1,630 | 0,930-2,868 | 0,080 | |  | | 1,340 | 0,635-2,841 | 0,439 |  |
| Lymphatic metastasis  *Negative*  *Positive* | 2,420 | 1,411-4,161 | 0,001 | |  | | 3,350 | 0,410-27,334 | 0,258 |  |
| SPARC expression  *<14,45*  *>14,45* | 1,680 | 0,964-2,933 | 0,060 | |  | | 1,350 | 0,715-2,547 | 0,350 |  |
| **Disease-free survival** |  |  |  | |  | |  |  |  |  |
| Age  *<70 years*  *>70 years* | 1,100 | 0,682-1,796 | 0,678 | |  | | 1,140 | 0,598-2,197 | 0,680 |  |
| Stage  *Early (1-2)*  *Advanced (3)* | 2,100 | 1,293-3,416 | 0,002 | |  | | 3,760 | 0,409-34,586 | 0,240 |  |
| Tumor size  *T1-2*  *T3-4* | 2,220 | 1,102-4,502 | 0,020 | |  | | 1,360 | 0,588-3,162 | 0,469 |  |
| Vascular invasion  *Negative*  *Positive* | 1,470 | 0,825-2,622 | 0,190 | |  | | 1,040 | 0,508-2,168 | 0,895 |  |
| Lymphovascular invasion  *Negative*  *Positive* | 1,650 | 0,997-2,732 | 0,050 | |  | | 1,630 | 0,802-3,336 | 0,175 |  |
| Lymphatic metastasis  *Negative*  *Positive* | 2,220 | 1,382-3,595 | 0,001 | |  | | 6,440 | 0,747-55,591 | 0,090 |  |
| SPARC expression  *<14,28*  *>14,28* | 1,590 | 0,956-2,650 | 0,070 | |  | | 1,330 | 0,730-2,436 | 0,348 |  |

**Supplementary Table S9. The prognostic significance of SPARC mRNA levels in patients with colon cancer revealed by univariate and multivariate COX analysis**

|  | Univariate | | | |  | | | Multivariate | | |
| --- | --- | --- | --- | --- | --- | --- | --- | --- | --- | --- |
| Parameter | HR | 95% CI | P-value | |  | | | HR | 95% CI | P-value |
| **Overall survival** |  |  |  | |  | | |  |  |  |
| Age  *<70 years*  *>70 years* | 2,760 | 1,458-5,234 | | 0,001 | |  | 3,320 | | 1,599-6,932 | 0,001 |
| Stage  *Early (1-2)*  *Advanced (3)* | 2,080 | 1,119-3,877 | | 0,020 | |  | 0,520 | | 0,55-4,753 | 0,558 |
| Tumor size  *T1-2*  *T3-4* | 2,290 | 0,899-5861 | | 0,810 | |  | 1,610 | | 0,554-4,716 | 0,378 |
| Vascular invasion  *Negative*  *Positive* | 1,330 | 0,670-2,662 | | 0,409 | |  | 0,960 | | 0,436-2,148 | 0,937 |
| Lymphovascular invasion  *Negative*  *Positive* | 2,210 | 1,184-4,127 | | 0,013 | |  | 1,600 | | 0,692-3,728 | 0,268 |
| Lymphatic metastasis  *Negative*  *Positive* | 2,390 | 1,314-4,367 | | 0,004 | |  | 4,600 | | 0,531-39,941 | 0,165 |
| SPARC expression  *<14,45*  *>14,45* | 1,550 | 0,843-2,878 | | 0,156 | |  | 1,180 | | 0,593-2,350 | 0,635 |
| **Disease-specific survival** |  |  | |  | |  |  | |  |  |
| Age  *<70 years*  *>70 years* | 1,740 | 0,745-3,988 | | 0,202 | |  | 1,360 | | 0,484-3,800 | 0,557 |
| Stage  *Early (1-2)*  *Advanced (3)* | 0,620 | 0,231-1,703 | | 0,361 | |  | 5,960 | | Invalid | 0,760 |
| Tumor size  *T1-2*  *T3-4* | 1,110 | 0,413-3,010 | | 0,831 | |  | 2,490 | | 0,556-11,200 | 0,232 |
| Vascular invasion  *Negative*  *Positive* | 1,530 | 0,5447-4,333 | | 0,415 | |  | 1,560 | | 0,489-5,000 | 0,449 |
| Lymphovascular invasion  *Negative*  *Positive* | 2,220 | 0,943-5,241 | | 0,067 | |  | 1,660 | | 0,553-5,000 | 0,365 |
| Lymphatic metastasis  *Negative*  *Positive* | 0,740 | 0,291-1,878 | | 0,526 | |  | 0,060 | | 0,000-5960 | 0,634 |
| SPARC expression  *<14,74*  *>14,74* | 2,640 | 1,141-6,110 | | 0,023 | |  | 7,440 | | 2,082-26,600 | 0,002 |
| **Disease-free survival** |  |  | |  | |  |  | |  |  |
| Age  *<70 years*  *>70 years* | 1,300 | 0,754-2,241 | | 0,344 | |  | 1,470 | | 0,767-2,827 | 0,244 |
| Stage  *Early (1-2)*  *Advanced (3)* | 1,820 | 1,0400-3,185 | | 0,035 | |  | 0,310 | | 0,034-2,752 | 0,291 |
| Tumor size  *T1-2*  *T3-4* | 2,190 | 0,895-4,867 | | 0,054 | |  | 1,730 | | 0,661-4,541 | 0,263 |
| Vascular invasion  *Negative*  *Positive* | 1,680 | 0,896-3,151 | | 0,105 | |  | 1,220 | | 0,585-2,554 | 0,592 |
| Lymphovascular invasion  *Negative*  *Positive* | 1,790 | 1,000-3,206 | | 0,049 | |  | 1,380 | | 0,632-3,043 | 0,413 |
| Lymphatic metastasis  *Negative*  *Positive* | 1,990 | 1,154-3,441 | | 0,013 | |  | 5,230 | | 0,637-43,026 | 0,123 |
| SPARC expression  *<14,28*  *>14,28* | 0,570 | 0,317-1,033 | | 0,064 | |  | 1,590 | | 0,789-3191 | 0,194 |
| **Progression-free survival** |  |  | |  | |  |  | |  |  |
| Age  *<70 years*  *>70 years* | 1,450 | 0,867-2,449 | | 0,154 | |  | 1,400 | | 0,801-2,453 | 0,235 |
| Stage  *Early (1-2)*  *Advanced (3)* | 0,890 | 0,514-1,550 | | 0,689 | |  | 9,640 | | 0,016-5705 | 0,486 |
| Tumor size  *T1-2*  *T3-4* | 1,370 | 0,713-2,663 | | 0,339 | |  | 1,850 | | 0,859-3,985 | 0,115 |
| Vascular invasion  *Negative*  *Positive* | 1,760 | 0,983-3,155 | | 0,056 | |  | 1,700 | | 0,909-3,188 | 0,095 |
| Lymphovascular invasion  *Negative*  *Positive* | 1,180 | 0,684-2,050 | | 0,544 | |  | 1,370 | | 0,716-2,646 | 0,336 |
| Lymphatic metastasis  *Negative*  *Positive* | 0,870 | 0,400-1,519 | | 0,636 | |  | 0,060 | | 0,000-41,470 | 0,414 |
| SPARC expression  *<14,52*  *>14,52* | 1,980 | 1,172-3,360 | | 0,010 | |  | 1,830 | | 1,021-3,302 | 0,042 |

**Supplementary Table S10. The prognostic significance of SPARC mRNA levels in patients with rectal cancer revealed by univariate and multivariate COX analysis**

|  | Univariate | | |  | Multivariate | | | |
| --- | --- | --- | --- | --- | --- | --- | --- | --- |
| Parameter | HR | 95% CI | P-value |  | HR | 95% CI | P-value |  |
| **Overall survival** |  |  |  |  |  |  |  |  |
| Age  *<70 years*  *>70 years* | 0,480 | 0,108-2,153 | 0,339 |  | 6,702 | 0,953-47,138 | 0,056 |  |
| Stage  *Early (1-2)*  *Advanced (3)* | 4,720 | 1,630-13,650 | 0,004 |  | Invalid | | | |
| Tumor size  *T1-2*  *T3-4* | 2,750 | 0,620-12,265 | 0,182 |  | 0,329 | 0,040-2,711 | 0,301 |  |
| Vascular invasion  *Negative*  *Positive* | 0,743 | 0,160-3,449 | 0,705 |  | 2,272 | 0,416-12,409 | 0,343 |  |
| Lymphovascular invasion  *Negative*  *Positive* | 1,370 | 0,487-3,880 | 0,547 |  | 0,445 | 0,043-4,525 | 0,494 |  |
| Lymphatic metastasis  *Negative*  *Positive* | 4,540 | 1,560-13,170 | 0,005 |  | 1,975 | 0,175-22,207 | 0,581 |  |
| SPARC expression  *<14,89*  *>14,89* | 5,840 | 1,404-21,421 | 0,014 |  | 6,356 | 0,951-42,459 | 0,056 |  |
| **Disease-specific survival** |  |  |  |  |  |  |  |  |
| Age  *<70 years*  *>70 years* | 0,480 | 0,108-2,153 | 0,339 |  | Invalid | | | |
| Stage  *Early (1-2)*  *Advanced (3)* | 4,720 | 1,630-13,650 | 0,004 |  | Invalid | | | |
| Tumor size  *T1-2*  *T3-4* | 2,750 | 0,620-12,265 | 0,182 |  | Invalid | | | |
| Vascular invasion  *Negative*  *Positive* | 0,743 | 0,160-3,449 | 0,705 |  | Invalid | | | |
| Lymphovascular invasion  *Negative*  *Positive* | 1,370 | 0,487-3,880 | 0,547 |  | Invalid | | | |
| Lymphatic metastasis  *Negative*  *Positive* | 4,540 | 1,560-13,170 | 0,005 |  | Invalid | | | |
| SPARC expression  *<13,97*  *>13,97* | 2,060 | 0,289-19,261 | 0,422 |  | Invalid | | | |
| **Disease-free survival** |  |  |  |  |  |  |  |  |
| Age  *<70 years*  *>70 years* | 0,480 | 0,108-2,153 | 0,339 |  | 0,378 | 0,067-2,110 | 0,268 |  |
| Stage  *Early (1-2)*  *Advanced (3)* | 4,720 | 1,630-13,650 | 0,004 |  | Invalid | | | |
| Tumor size  *T1-2*  *T3-4* | 2,750 | 0,620-12,265 | 0,182 |  | 1,985 | 0,305-12,922 | 0,427 |  |
| Vascular invasion  *Negative*  *Positive* | 0,743 | 0,160-3,449 | 0,705 |  | 0,266 | 0,042-1,673 | 0,158 |  |
| Lymphovascular invasion  *Negative*  *Positive* | 1,370 | 0,487-3,880 | 0,547 |  | 0,658 | 0,126-3,417 | 0,618 |  |
| Lymphatic metastasis  *Negative*  *Positive* | 4,540 | 1,560-13,170 | 0,005 |  | 6,545 | 1,145-37,141 | 0,034 |  |
| SPARC expression  *<14,86*  *>14,86* | 2,830 | 1,019-7,907 | 0,045 |  | 2,383 | 0,616-9,212 | 0,207 |  |
| **Progression-free survival** |  |  |  |  |  |  |  |  |
| Age  *<70 years*  *>70 years* | 0,480 | 0,108-2,153 | 0,339 |  | Invalid | | | |
| Stage  *Early (1-2)*  *Advanced (3)* | 4,720 | 1,630-13,650 | 0,004 |  | Invalid | | | |
| Tumor size  *T1-2*  *T3-4* | 2,750 | 0,620-12,265 | 0,182 |  | Invalid | | | |
| Vascular invasion  *Negative*  *Positive* | 0,743 | 0,160-3,449 | 0,705 |  | Invalid | | | |
| Lymphovascular invasion  *Negative*  *Positive* | 1,370 | 0,487-3,880 | 0,547 |  | Invalid | | | |
| Lymphatic metastasis  *Negative*  *Positive* | 4,540 | 1,560-13,170 | 0,005 |  | Invalid | | | |
| SPARC expression  *<14,25*  *>14,25* | 4,504 | 1,332-15,225 | 0,015 |  | Invalid | | | |

**Supplementary Table S11. The prognostic significance of SPP1 mRNA levels in patients with CRC revealed by univariate and multivariate COX analysis**

|  | Univariate | | |  | Multivariate | | |
| --- | --- | --- | --- | --- | --- | --- | --- |
| Parameter | HR | 95% CI | P-value |  | HR | 95% CI | P-value |
| **Overall survival** |  |  |  |  |  |  |  |
| Age  *<70 years*  *>70 years* | 3,200 | 1,811-5,667 | <0,0001 |  | 3,560 | 1,848-6,790 | 0,0001 |
| Stage  *Early (1-2)*  *Advanced (3)* | 2,170 | 1,234-3,840 | 0,007 |  | 0,640 | 0,074-5,473 | 0,683 |
| Tumor size  *T1-2*  *T3-4* | 2,050 | 0,926-4,575 | 0,070 |  | 1,270 | 0,514-3,144 | 0,602 |
| Vascular invasion  *Negative*  *Positive* | 1,420 | 0,766-2,639 | 0,263 |  | 1,270 | 0,632-2,558 | 0,498 |
| Lymphovascular invasion  *Negative*  *Positive* | 1,630 | 0,930-2,868 | 0,080 |  | 1,320 | 0,626-2,805 | 0,461 |
| Lymphatic metastasis  *Negative*  *Positive* | 2,420 | 1,411-4,161 | 0,001 |  | 3,420 | 0,411-28,375 | 0,254 |
| SPP1 expression  *<11,65*  *>11,65* | 1,660 | 0,957-2,884 | 0,070 |  | 1,150 | 0,599-2,241 | 0,660 |
| **Disease-specific survival** |  |  |  |  |  |  |  |
| Age  *<70 years*  *>70 years* | 1,570 | 0,775-3,199 | 0,208 |  | 1,320 | 0,533-3,300 | 0,547 |
| Stage  *Early (1-2)*  *Advanced (3)* | 2,620 | 1,001-6,894 | 0,0049 |  | 3,120 | Invalid | 0,832 |
| Tumor size  *T1-2*  *T3-4* | 1,670 | 0,642-4,364 | 0,291 |  | 3,300 | 0,755-14,400 | 0,112 |
| Vascular invasion  *Negative*  *Positive* | 2,420 | 1,067-5,496 | 0,034 |  | 1,760 | 0,661-4,700 | 0,257 |
| Lymphovascular invasion  *Negative*  *Positive* | 1,170 | 0,549-2,507 | 0,679 |  | 1,030 | 0,394-3,100 | 0,856 |
| Lymphatic metastasis  *Negative*  *Positive* | 2,240 | 0,917-5,502 | 0,228 |  | 0,090 | 0-3841 | 0,667 |
| SPP1 expression  *<10,94*  *>10,94* | 1,520 | 0,751-3,077 | 0,243 |  | 1,930 | 0,778-4,800 | 0,156 |
| **Disease-free survival** |  |  |  |  |  |  |  |
| Age  *<70 years*  *>70 years* | 1,100 | 0,682-1,796 | 0,678 |  | 1,250 | 0,702-2,228 | 0,446 |
| Stage  *Early (1-2)*  *Advanced (3)* | 2,100 | 1,293-3,416 | 0,002 |  | 0,250 | 0,029-2,153 | 0,209 |
| Tumor size  *T1-2*  *T3-4* | 2,220 | 1,102-4,502 | 0,020 |  | 1,570 | 0,683-3,621 | 0,286 |
| Vascular invasion  *Negative*  *Positive* | 1,470 | 0,825-2,622 | 0,190 |  | 1,120 | 0,576-2,171 | 0,740 |
| Lymphovascular invasion  *Negative*  *Positive* | 1,650 | 0,997-2,732 | 0,050 |  | 1,310 | 0,674-2,568 | 0,421 |
| Lymphatic metastasis  *Negative*  *Positive* | 2,220 | 1,382-3,595 | 0,001 |  | 7,10 | 0,865-58,394 | 0,067 |
| SPP1 expression  *<11,60*  *>11,60* | 1,690 | 1,044-2,6745 | 0,032 |  | 1,230 | 0,702-2,342 | 0,417 |
| **Progression-free survival** |  |  |  |  |  |  |  |
| Age  *<70 years*  *>70 years* | 1,370 | 0,889-2,139 | 0,150 |  | 1,440 | 0,795-2,324 | 0,131 |
| Stage  *Early (1-2)*  *Advanced (3)* | 0,840 | 0,527-1,340 | 0,467 |  | 7,440 | 0,022-2501,815 | 0,498 |
| Tumor size  *T1-2*  *T3-4* | 1,310 | 0,758-2,276 | 0,330 |  | 1,870 | 0,968-3,628 | 0,062 |
| Vascular invasion  *Negative*  *Positive* | 1,610 | 0,962-2,697 | 0,069 |  | 1,500 | 0,877-2,501 | 0,136 |
| Lymphovascular invasion  *Negative*  *Positive* | 1,250 | 0,793-1978 | 0,333 |  | 1,580 | 0,911-2,769 | 0,102 |
| Lymphatic metastasis  *Negative*  *Positive* | 0,820 | 0,517-1,315 | 0,118 |  | 0,070 | 0,0002-26,577 | 0,391 |
| SPP1 expression  *<10,51*  *>10,51* | 2,200 | 1,382-3,526 | 0,0009 |  | 2,510 | 1,493-4,225 | 0,0005 |

**Supplementary Table S12. The prognostic significance of SPP1 mRNA levels in patients with colon cancer revealed by univariate and multivariate COX analysis**

|  | Univariate | | |  | Multivariate | | |
| --- | --- | --- | --- | --- | --- | --- | --- |
| Parameter | HR | 95% CI | P-value |  | HR | 95% CI | P-value |
| **Overall survival** |  |  |  |  |  |  |  |
| Age  *<70 years*  *>70 years* | 2,760 | 1,458-5,234 | 0,001 |  | 3,590 | 1,716-7,545 | <0,0001 |
| Stage  *Early (1-2)*  *Advanced (3)* | 2,080 | 1,119-3,877 | 0,020 |  | 0,390 | 0,041-3,752 | 0,416 |
| Tumor size  *T1-2*  *T3-4* | 2,290 | 0,899-5861 | 0,810 |  | 1,620 | 0,953-4,751 | 0,377 |
| Vascular invasion  *Negative*  *Positive* | 1,330 | 0,670-2,662 | 0,409 |  | 0,890 | 0,401-2,000 | 0,789 |
| Lymphovascular invasion  *Negative*  *Positive* | 2,210 | 1,184-4,127 | 0,013 |  | 1,740 | 0,743-4,091 | 0,201 |
| Lymphatic metastasis  *Negative*  *Positive* | 2,390 | 1,314-4,367 | 0,004 |  | 5,890 | 0,774-51,558 | 0,108 |
| SPP1 expression  *<11,65*  *>11,65* | 2,120 | 1,092-4,149 | 0,026 |  | 1,820 | 0,828-4,007 | 0,135 |
| **Disease-specific survival** |  |  |  |  |  |  |  |
| Age  *<70 years*  *>70 years* | 1,740 | 0,745-3,988 | 0,202 |  | 1,690 | 0,604-4,750 | 0,315 |
| Stage  *Early (1-2)*  *Advanced (3)* | 0,620 | 0,231-1,703 | 0,361 |  | 2,930 | Invalid | 0,835 |
| Tumor size  *T1-2*  *T3-4* | 1,110 | 0,413-3,010 | 0,831 |  | 2,200 | 0,493-9,810 | 0,301 |
| Vascular invasion  *Negative*  *Positive* | 1,530 | 0,544-4,333 | 0,415 |  | 1,370 | 0,426-4,410 | 0,595 |
| Lymphovascular invasion  *Negative*  *Positive* | 2,220 | 0,943-5,241 | 0,067 |  | 1,540 | 0,448-4,760 | 0,452 |
| Lymphatic metastasis  *Negative*  *Positive* | 0,740 | 0,291-1,878 | 0,526 |  | 0,160 | 0,000-4488 | 0,730 |
| SPP1 expression  *<10,94*  *>10,94* | 1,090 | 0,483-2,489 | 0,823 |  | 1,280 | 0,464-3,550 | 0,630 |
| **Disease-free survival** |  |  |  |  |  |  |  |
| Age  *<70 years*  *>70 years* | 1,300 | 0,754-2,241 | 0,344 |  | 1,510 | 0,787-2,900 | 0,140 |
| Stage  *Early (1-2)*  *Advanced (3)* | 1,820 | 1,040-3,185 | 0,035 |  | 0,310 | 0,034-2,743 | 0,291 |
| Tumor size  *T1-2*  *T3-4* | 2,190 | 0,895-4,867 | 0,054 |  | 1,750 | 0,668-4,616 | 0,252 |
| Vascular invasion  *Negative*  *Positive* | 1,680 | 0,896-3,151 | 0,105 |  | 1,260 | 0,604-2,635 | 0,535 |
| Lymphovascular invasion  *Negative*  *Positive* | 1,790 | 1,000-3,206 | 0,049 |  | 1,350 | 0,620-2,953 | 0,447 |
| Lymphatic metastasis  *Negative*  *Positive* | 1,990 | 1,154-3,441 | 0,013 |  | 5,360 | 0,642-4,838 | 0,120 |
| SPP1 expression  *<11,60*  *>11,60* | 1,630 | 0,942-2,835 | 0,080 |  | 1,220 | 0,624-2,386 | 0,559 |
| **Progression-free survival** |  |  |  |  |  |  |  |
| Age  *<70 years*  *>70 years* | 1,450 | 0,867-2,449 | 0,154 |  | 1,450 | 0,834-2,547 | 0,185 |
| Stage  *Early (1-2)*  *Advanced (3)* | 0,890 | 0,514-1,550 | 0,689 |  | 10,730 | 0,013-8756 | 0,487 |
| Tumor size  *T1-2*  *T3-4* | 1,370 | 0,713-2,663 | 0,339 |  | 1,830 | 0,853-3,943 | 0,120 |
| Vascular invasion  *Negative*  *Positive* | 1,760 | 0,983-3,155 | 0,056 |  | 1,640 | 0,882-3,053 | 0,117 |
| Lymphovascular invasion  *Negative*  *Positive* | 1,180 | 0,684-2,050 | 0,544 |  | 1,530 | 0,798-2,954 | 0,198 |
| Lymphatic metastasis  *Negative*  *Positive* | 0,870 | 0,400-1,519 | 0,636 |  | 0,060 | 0,000-49,947 | 0,413 |
| SPP1 expression  *<10,51*  *>10,51* | 2,080 | 1,212-3,585 | 0,007 |  | 2,350 | 1,294-4,283 | 0,005 |

**Supplementary Table S13. The prognostic significance of SPP1 mRNA levels in patients with rectal cancer revealed by univariate and multivariate COX analysis**

|  | Univariate | | |  | Multivariate | | |
| --- | --- | --- | --- | --- | --- | --- | --- |
| Parameter | HR | 95% CI | P-value |  | HR | 95% CI | P-value |
| **Overall survival** |  |  |  |  |  |  |  |
| Age  *<70 years*  *>70 years* | 0,480 | 0,108-2,153 | 0,339 |  | 9,628 | 1,056-87,716 | 0,044 |
| Stage  *Early (1-2)*  *Advanced (3)* | 4,720 | 1,630-13,650 | 0,004 |  | Invalid | | |
| Tumor size  *T1-2*  *T3-4* | 2,750 | 0,620-12,265 | 0,182 |  | 0,868 | 0,115-6,539 | 0,891 |
| Vascular invasion  *Negative*  *Positive* | 0,743 | 0,160-3,449 | 0,705 |  | 3,731 | 0,654-21,259 | 0,138 |
| Lymphovascular invasion  *Negative*  *Positive* | 1,370 | 0,487-3,880 | 0,547 |  | 1,191 | 0,132-10,676 | 0,875 |
| Lymphatic metastasis  *Negative*  *Positive* | 4,540 | 1,560-13,170 | 0,005 |  | 0,960 | 0,092-10,001 | 0,973 |
| SPP1 expression  *<13,19*  *>13,19* | 0,534 | 0,113-2,520 | 0,428 |  | 10,292 | 0,521-203,124 | 0,125 |
| **Disease-specific survival** |  |  |  |  |  |  |  |
| Age  *<70 years*  *>70 years* | 0,480 | 0,108-2,153 | 0,339 |  | Invalid | | |
| Stage  *Early (1-2)*  *Advanced (3)* | 4,720 | 1,630-13,650 | 0,004 |  | Invalid | | |
| Tumor size  *T1-2*  *T3-4* | 2,750 | 0,620-12,265 | 0,182 |  | Invalid | | |
| Vascular invasion  *Negative*  *Positive* | 0,743 | 0,160-3,449 | 0,705 |  | Invalid | | |
| Lymphovascular invasion  *Negative*  *Positive* | 1,370 | 0,487-3,880 | 0,547 |  | Invalid | | |
| Lymphatic metastasis  *Negative*  *Positive* | 4,540 | 1,560-13,170 | 0,005 |  | Invalid | | |
| SPP1 expression  *<11,48*  *>11,48* | 2,980 | 0,709-12,580 | 0,135 |  | Invalid | | |
| **Disease-free survival** |  |  |  |  |  |  |  |
| Age  *<70 years*  *>70 years* | 0,480 | 0,108-2,153 | 0,339 |  | 0,244 | 0,040-1,488 | 0,126 |
| Stage  *Early (1-2)*  *Advanced (3)* | 4,720 | 1,630-13,650 | 0,004 |  | Invalid | | |
| Tumor size  *T1-2*  *T3-4* | 2,750 | 0,620-12,265 | 0,182 |  | 1,824 | 0,284-11,707 | 0,526 |
| Vascular invasion  *Negative*  *Positive* | 0,743 | 0,160-3,449 | 0,705 |  | 0,268 | 0,044-1,640 | 0,154 |
| Lymphovascular invasion  *Negative*  *Positive* | 1,370 | 0,487-3,880 | 0,547 |  | 0,752 | 0,163-3,468 | 0,714 |
| Lymphatic metastasis  *Negative*  *Positive* | 4,540 | 1,560-13,170 | 0,005 |  | 5,718 | 0,973-33,587 | 0,053 |
| SPP1 expression  *<11,37*  *>11,37* | 2,805 | 1,016-7,741 | 0,046 |  | Invalid | | |
| **Progression-free survival** |  |  |  |  |  |  |  |
| Age  *<70 years*  *>70 years* | 0,480 | 0,108-2,153 | 0,339 |  | 1,450 | 0,541-3,895 | 0,458 |
| Stage  *Early (1-2)*  *Advanced (3)* | 4,720 | 1,630-13,650 | 0,004 |  | Invalid | | |
| Tumor size  *T1-2*  *T3-4* | 2,750 | 0,620-12,265 | 0,182 |  | 2,420 | 0,626-9,390 | 0,199 |
| Vascular invasion  *Negative*  *Positive* | 0,743 | 0,160-3,449 | 0,705 |  | 1,14 | 0,337-3,389 | 0,827 |
| Lymphovascular invasion  *Negative*  *Positive* | 1,370 | 0,487-3,880 | 0,547 |  | 1,57 | 0,510-4,862 | 0,428 |
| Lymphatic metastasis  *Negative*  *Positive* | 4,540 | 1,560-13,170 | 0,005 |  | 0,370 | 0,110-0,126 | 0,119 |
| SPP1 expression  *<10,59*  *>10,59* | 2,780 | 1,089-7,125 | 0,032 |  | 3,320 | 1,124-9,809 | 0,029 |
